# Effect of Ionic Diffusion on Extracellular Potentials in Neural Tissue

**DOI:** 10.1371/journal.pcbi.1005193

**Published:** 2016-11-07

**Authors:** Geir Halnes, Tuomo Mäki-Marttunen, Daniel Keller, Klas H. Pettersen, Ole A. Andreassen, Gaute T. Einevoll

**Affiliations:** 1 Department of Mathematical Sciences and Technology, Norwegian University of Life Sciences, Ås, Norway; 2 NORMENT, KG Jebsen Centre for Psychosis Research, Institute of Clinical Medicine, University of Oslo, Oslo, Norway; 3 Blue Brain Project, École Polytechnique Fédérale de Lausanne (EPFL), Lausanne, Switzerland; 4 Letten Centre and GliaLab, Institute of Basic Medical Sciences, University of Oslo, Oslo, Norway; 5 Centre for Molecular Medicine Norway, University of Oslo, Oslo, Norway; 6 Department of Physics, University of Oslo, Oslo, Norway; George Mason University, UNITED STATES

## Abstract

Recorded potentials in the extracellular space (ECS) of the brain is a standard measure of population activity in neural tissue. Computational models that simulate the relationship between the ECS potential and its underlying neurophysiological processes are commonly used in the interpretation of such measurements. Standard methods, such as volume-conductor theory and current-source density theory, assume that diffusion has a negligible effect on the ECS potential, at least in the range of frequencies picked up by most recording systems. This assumption remains to be verified. We here present a hybrid simulation framework that accounts for diffusive effects on the ECS potential. The framework uses (1) the NEURON simulator to compute the activity and ionic output currents from multicompartmental neuron models, and (2) the electrodiffusive Kirchhoff-Nernst-Planck framework to simulate the resulting dynamics of the potential and ion concentrations in the ECS, accounting for the effect of electrical migration as well as diffusion. Using this framework, we explore the effect that ECS diffusion has on the electrical potential surrounding a small population of 10 pyramidal neurons. The neural model was tuned so that simulations over ∼100 seconds of biological time led to shifts in ECS concentrations by a few millimolars, similar to what has been seen in experiments. By comparing simulations where ECS diffusion was absent with simulations where ECS diffusion was included, we made the following key findings: (i) ECS diffusion shifted the local potential by up to ∼0.2 mV. (ii) The power spectral density (PSD) of the diffusion-evoked potential shifts followed a 1/*f*^2^ power law. (iii) Diffusion effects dominated the PSD of the ECS potential for frequencies up to several hertz. In scenarios with large, but physiologically realistic ECS concentration gradients, diffusion was thus found to affect the ECS potential well within the frequency range picked up in experimental recordings.

## Author Summary

When electrical potentials are measured in the extracellular space (ECS) of the brain, they are interpreted as a signature of neural signalling. The relationship between the ECS potentials and the underlying neuronal processes is often studied with the aid of computer models. The ECS potential is typically assumed not to be affected by diffusive currents in the ECS, and existing models therefore neglect diffusion. However, there may be scenarios where this assumption does not hold. Here, we present a new computational model which explicitly models ion-concentration dynamics in the ECS surrounding a neural population, and which allows us to quantify the effect that diffusive currents have on the ECS potential. Using this model, we simulate a scenario where a population of pyramidal neurons is active over a long time, and produces large, but realistic concentration gradients in the ECS. In this scenario, diffusive currents are found to influence the ECS potential at frequency components as high as ten hertz. Unlike previously believed, we thus predict that there are scenarios where recorded local field potentials (LFPs) are likely to contain signatures not only of neural activity, but also of ECS diffusion.

## Introduction

The number of ions exchanged between neurons and the extracellular space (ECS) during a brief period of activity (i.e., due to the integration of synaptic input and generation of a few action potentials) is typically too small to evoke significant changes in extracellular ion concentrations. In models of short-term electrical signalling of neurons, the ion concentrations of the main charge carriers (e.g., K^+^, Na^+^, Cl^-^) are therefore commonly assumed to remain effectively constant. This assumption often holds also at longer time scales, due to the work done by neuronal and glial uptake mechanisms in maintaining ion concentrations close to baseline levels. However, during periods of intense neural signalling, the uptake mechanisms may fail to keep up, and ion concentrations in the ECS may change by several millimolars [1–5]. For example, the extracellular K^+^ concentration can increase from a typical baseline level of around 3 mM and up to levels between 8 and 12 mM during non-pathological conditions [4, 6–8]. Ion-concentration shifts in the ECS will change neuronal reversal potentials and firing patterns [9–12], and too large deviations from baseline levels can lead to pathological conditions such as hypoxia, anoxia, ischemia, epilepsy and spreading depression [9, 13–15].

One of the most common experimental methods for investigating neural activity is the measurement of electrical potentials with extracellular electrodes. Commonly, it is assumed that extracellular potentials predominantly reflect transmembrane cellular current sources, including synaptic currents and currents through active and passive membrane mechanisms in neurons and glial cells [16–19]. However, in scenarios where ECS concentration gradients become sufficiently large, electrical currents carried by diffusing ions in the ECS could in principle also give measurable effects on the extracellular electrical potentials (cf., liquid junction potentials [20–22]). In support of this, local ion-concentration changes in the ECS are indeed often accompanied by slow local negative potential shifts, which can be on the order of a few millivolts [1, 3, 13, 23–27]. Whereas K^+^ buffering currents through the glia-cell membranes are believed to be the main source of these slow potential shifts [3, 7], it has been estimated that also diffusive currents along extracellular concentration gradients could contribute by shifting ECS potentials by up to 0.4 mV [3]. As ion concentrations in the ECS typically vary on the time scale of seconds [3, 4, 28], it is nevertheless *a priori* unclear whether diffusion-evoked potential shifts would be picked up by the electrode measurement systems applied in most experiments, which typically have cut-off frequencies of about 0.1–0.2 Hz or higher (see e.g., [29, 30]).

In most computational studies of ECS potentials, diffusive currents in the ECS are assumed to be negligible compared to the currents propelled by the electrical field (hereby termed field currents). This is, for example, an underlying assumption in volume-conductor theory which has been the basis for estimating ECS potentials from cellular current sources [18, 31–36], and in estimation of current-source density (CSD) which predicts transmembrane neural current sources from recordings of extracellular potentials [29, 34, 37–40]. Another series of theoretical studies have aimed to incorporate possible effects of diffusion in the complex impedance environment of the extracellular medium [41–44], and have suggested that such effects may account for the 1/*f*-scaling observed for the LFP-power spectrum at low frequencies [41]. In neither of the above mentioned studies, however, ionic diffusion was explicitly modelled.

The reason why diffusive effects are often neglected in models of extracellular fields, may be that the task of modelling it is challenging. This is because the study of diffusion requires an explicit tracking of all present ions and their spatiotemporal dynamics: i.e., keeping track of only the electric currents and net electric charges is not sufficient. Existing electrodiffusive models have typically been based on the Poisson-Nernst-Planck (PNP) formalism [45–51]. The PNP formalism explicitly models charge-relaxation processes, which occur at spatiotemporal scales on the order of nanometers and nanoseconds. This requires an extremely high spatiotemporal resolution, which makes PNP models computationally expensive and unsuited for predictions at the tissue/population level [52]. However, a series of modelling schemes have been developed that circumvent the charge relaxation processes, essentially by replacing Poisson’s equation by the constraint that the bulk solution is electroneutral [28, 52–58]. The electroneutrality condition is a physical constraint valid at a larger spatiotemporal scale, and thus allows for a dramatic increase in the spatial and temporal grid sizes in the numerical simulations. One of these simpler models were previously developed by our group [28, 57], and is here referred to as the *Kirchhoff-Nernst-Planck (KNP)* scheme. The KNP scheme is a means of deriving the local potential in the intra- and extracellular bulk solution from the constraint that Kirchhoff’s current law should be fulfilled for all finite volumes (the sum of currents into a finite subvolume of bulk solution should be zero).

**Fig 1:**
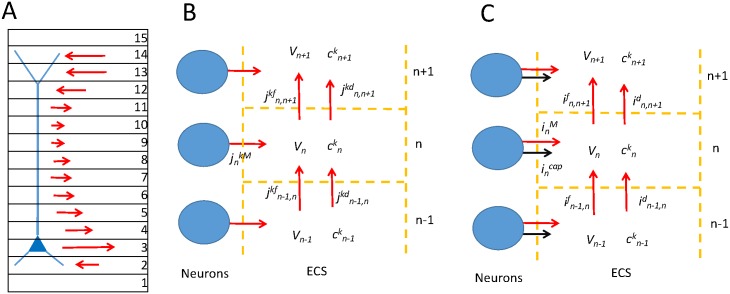
Model system. **(A)** A piece of neural tissue was subdivided into 15 subvolumes (depth intervals). The edges *n* = 1 and *n* = 15 were auxiliary compartments used to implement appropriate boundary conditions. In these subvolumes ion concentrations were set to be constant baseline levels. In *n* = 1, the ECS potential was set to *V* = 0, while in *n* = 15, *V* was derived so that no net current entered/left the system (see Methods for details). A population of 10 neurons (only one shown in the figure) was positioned so that it occupied the interior 13 subvolumes. The output of specific ions into each subvolume was computed for all segments of all 10 neurons and summed, yielding the total input of an ion species *k* to each subvolume (illustrated by red arrows). **(B)** Ion-concentration dynamics in an ECS subvolume *n*. Here $j_{n}^{kM}$ denotes the total transmembrane flux density of ion species *k* into the subvolume *n* from the whole population of neurons. *j*^*kf*^ and *j*^*kd*^ denote ECS flux densities between neighboring subvolume driven by electrical potential differences and diffusion, respectively. **(C)** The extracellular potential is calculated by demanding that the sum of currents into each ECS subvolume is zero. Currents were determined by summing the contributions from all ionic fluxes (red arrows), and adding the capacitive current (black arrows).

In the current work we have developed a hybrid modelling formalism that allows us to compute electrodiffusive ion dynamics in the ECS surrounding active neurons. The formalism is briefly summarized in Fig 1. First, it utilizes the NEURON simulator [59, 60], which is a standard tool for simulating morphologically complex neurons, to simulate the activity of a neural population and its exchange of ions with the ECS (Fig 1A). Second, it utilizes the KNP formalism [28, 57] to compute the dynamics of ion concentrations and the electrical potential in the ECS surrounding the neurons (Fig 1B). The KNP scheme accounts for all electrical currents entering an ECS subvolume in the system (i.e., transmembrane ionic currents, transmembrane capacitive currents, diffusive currents through the ECS, and field currents through the ECS), as well as for concentration-dependent variations in the ECS conductivity (see Methods). It computes the ECS potential from the constraint that all currents into a ECS subvolume should sum to zero (Fig 1C). In this way, the KNP-scheme accounts for effects of ionic diffusion on the ECS potential, and thus differs from previous simulation schemes for computing ECS potentials based on output from standard neuron simulators such as NEURON (e.g. [61]).

We have here used the hybrid scheme to model a small tissue element consisting of a population of ten pyramidal neurons embedded in ECS (Fig 1A). Motivated by the layered structures of cortex and hippocampus, we assumed lateral homogeneity, so that all spatial variation occurred in the vertical direction. As neuronal model, we used a well established multicompartmental model of pyramidal cells [62]. The ten neurons were centered at the same depth level of the tissue. Although the choice of neuronal model was somewhat arbitrary, and the small tissue element was too simple to represent any particular biological system, the model gave rise to biologically realistic variations in ECS concentrations, and we regard it as a meaningful scenario for which we could explore how diffusive currents in the ECS can influence extracellular potentials.

In simulations that evoked large, but not pathologically large, concentration gradients in the ECS, we found that diffusion gave rise to a detectable 1/*f*^2^ power law in the low-frequency part of the power spectral density (PSD) of the ECS potential. Furthermore, we found that diffusion influenced the PSD for frequencies as high as 1–10 Hz. This quantitative prediction was, of course, specific to the particular model setup used here. Although the relative effects of diffusion may be smaller in many realistic, more complex scenarios (see Discussion), we regard our findings as an important demonstration that in general, diffusive currents can not by default be assumed to have a negligible impact on ECS potentials.

The article is organized as follows: In the Results section, we use the KNP scheme to explore the role of diffusive currents on electrical potentials in the ECS surrounding a population of pyramidal neurons. In the Discussion section, we discuss possible implications that our findings will have for the interpretation of data from extracellular recordings. The Discussion also includes an overview of the assumptions made in the presented model, and on how the framework can be expanded to allow for more thorough investigations of concentration-dependent effects on ion dynamics in neural tissue. A detailed derivation of the KNP-formalism is postponed to the Methods section (which is found at the end of the article).

## Results

The strategy employed in the current study was as follows: First, we simulated the neurodynamics of a small population of ten pyramidal neurons by means of the simulation tool NEURON, and recorded (as a time series) the transmembrane output of all ionic species, as well as the capacitive current, into the different subvolumes of the ECS (Fig 1A). For simplicity, we assumed that the neurodynamics was independent of the ECS dynamics. The simulation was run for a long time period (84 seconds), since ECS diffusion typically takes place on a much longer time scale than the millisecond time scale of neuronal firing and synaptic integration. Second, we used the KNP-formalism to simulate the ECS dynamics resulting from the neuronal output (Fig 1A and 1B). We considered the two cases where (i) diffusive transports were *not* included (i.e., so that ECS ion transports were solely due to field currents), and (ii) where diffusive transports were included. In the simulations the time-varying neuronal output was applied as an external input to the ECS system. An identical neuronal output was used in the two cases ((i) and (ii)). Third, we compared the ECS potential obtained in the two cases to demonstrate how it was affected by the inclusion of diffusion.

The simulation setup is briefly introduced in the following section, while further details are found in the Methods section. A list of symbols and definitions is given in Table 1.

**Table 1:** List of key symbols and constants.

Symbol	Explanation	Value/Unit
$J_{n}^{kM}$	Net membrane flux of ion *k* into subvolume *n*	mol/s
$I_{n}^{M}$	Net ionic membrane current into subvolume *n*	A
$I_{n}^{cap}$	Capacitive current into subvolume *n*	A
*V*_*n*_	Extracellular potential in subvolume *n*	V
$J_{n-1,n}^{kf}$	Electrical field flux of ion *k* from subvolume *n* − 1 to *n*	mol/s
$I_{n-1,n}^{f}$	Electrical field current from subvolume *n* − 1 to *n*	A
$J_{n-1,n}^{kd}$	Diffusive flux of ion *k* from subvolume *n* − 1 to *n*	mol/s
$I_{n-1,n}^{d}$	Diffusive current from subvolume *n* − 1 to *n*	A
*l*_*c*_	Height of each ECS subvolume box	100 *μ*m
*A*_*c*_	Cross-sectional area of each ECS subvolume box	600 *μ*m^2^
*c*_*K*0_	Baseline ECS K^+^ concentration	3 mM
*c*_*Na*0_	Baseline ECS Na^+^ concentration	150 mM
*c*_*Ca*0_	Baseline ECS Ca^2+^ concentration	1.4 mM
*c*_*X*0_	Baseline ECS X^-^ concentration	155.8 mM

### Dynamics of a small neuronal population

Ten pyramidal neurons were simulated by running ten independent simulations on a single neuron model. As neuron model, we used a well established model developed for cortical layer 5 pyramidal cells [62]. Each neuron was driven by uncorrelated Poissonian input spike trains (with the same statistics for all neurons) through 10,000 synapses. Synapses were uniformly distributed over the membrane area (sections with equal membrane area had the same expected number of synapses), and synaptic weights were tuned so that the average single-neuron action potential (AP) firing rate was about five APs per second (this is within the range of typical firing frequencies observed for cortical neurons [63]).

As illustrated in Fig 1, a piece of tissue was subdivided vertically into 15 depth intervals (here referred to as ECS subvolumes), which we could picture as spanning from the bottom to the top layer of a layered structure such as cortex or hippocampus. The neurons were positioned so that they occupied the 13 interior subvolumes. The output from all neural segments contained in a specific subvolume were summed, and this gave the total output into the given subvolume. In the neuronal output signal we kept separate track of the different kinds of transmembrane currents, including (i) the net Na^+^ current, (ii) the net K^+^ current, (iii) the net Ca^2+^ current, (iv) non-specific ionic currents, and (v) the capacitive current. For simplicity, we assumed that all unspecified ionic currents in the model [62] (such as leakage currents, synaptic currents, and currents through non-specific active ion channels) were carried by a single, non-specified anion species X^-^. We chose to use an anion, because many of the non-specified currents are likely to be mediated largely by Cl^-^ (for further comments on this choice, see Methods and Discussion).

**Fig 2:**
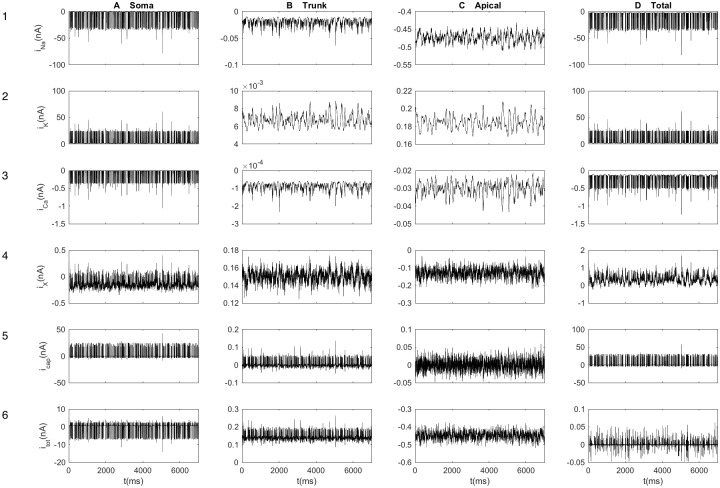
Output from the neuronal population. Transmembrane currents into selected extracellular volumes, including (column **A**) the subvolume containing the neuronal somata (*n* = 3), (column **B**) the subvolume containing the trunk of the apical dendrite (*n* = 7), and (column **C**) the subvolume where the apical dendrites branched out (*n* = 13). Currents were subdivided into ion specific currents (row **1**–**4**) and the capacitive current (row **5**). The sum of all currents into a subvolume *n* is shown in row **6**. The location of the midpoint of a neural segment determined which ECS subvolume *n* it belonged to, and currents were summed over all neural segments (of all neurons) that occupied a given ECS-subvolume (*n*). The transmembrane currents were defined as positive when crossing the membrane in the outward direction. The total transmembrane currents of the neuron as a whole (summed over all *N* − 2 subvolumes) were also calculated (column **D**). Results are shown for a 7 second excerpt of simulations.

The output from the neural population into three selected ECS subvolumes is shown in Fig 2 for the first seven seconds of the simulation. For example, Fig 2A shows the currents into the subvolume (*n* = 3) containing the somata. Here, we clearly see the brief Na^+^ (Fig 2A1) and K^+^ (Fig 2A2) current pulses associated with neuronal AP firing. The current amplitudes were about -30 nA (inward, depolarizing current) for Na^+^ and 30 nA (outward, repolarizing current) for K^+^. Generally, the subvolume containing the somata received a higher influx/efflux of ions (Fig 2A) compared to the subvolumes containing the apical trunk (Fig 2B) and apical branches (Fig 2C). These differences have two explanations: First, the somata subvolume contained a larger proportion of the total neuronal membrane area, which generally enhanced the ionic exchange in this subvolume. (Similarly, currents are larger in Fig 2C compared to Fig 2B because the subvolume where the apical dendrites branched out contained a larger membrane area than subvolumes containing a part of the apical dendritic trunk.) Secondly, the somata also had a higher density of Na^+^ and K^+^ channels than the dendrites. Accordingly, almost all exchange of Na^+^ and K^+^ between the neurons and the ECS occurred in the soma subvolume (compare somatic output in Fig 2A1 and 2A2 to the total neuronal output current in Fig 2D1 and 2D2). For the other ions (Ca^2+^ and X^-^), the dendrites contributed with a larger proportion of the total output.

**Fig 3:**
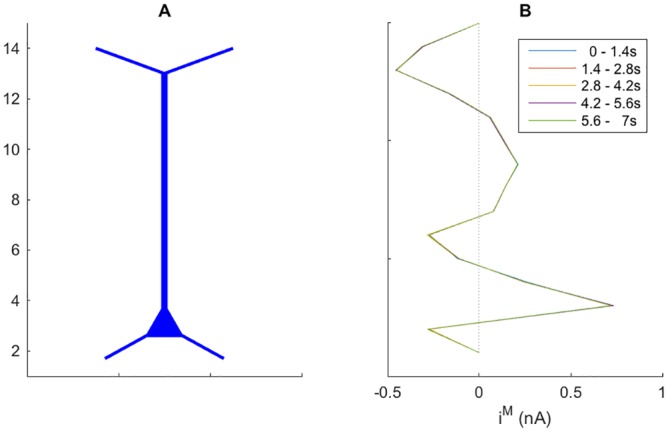
Transmembrane current profiles. **(A)** Tissue subdivided into 15 sub-volumes. **(B)** Distribution of *I*^*M*^ over the depth of the piece of tissue. *I*^*M*^ included all transmembrane currents (ionic + capacitive), and was low pass filtered by taking the temporal average over the time intervals indicated in the legend.

As we just saw, the neurodynamics fluctuated vividly on the millisecond time scale. However, the input statistics was the same throughout the simulation, so that the slow time-scale neurodynamics was essentially stationary (see Methods). To illustrate this, we split the seven seconds of neural simulations shown in Fig 2 into five 1.4 second time intervals, and averaged the total transmembrane current (*I*^*M*^) over the five respective intervals. Fig 3 shows how the (temporally averaged) transmembrane sources were distributed across tissue depth. The spatial profile of *I*^*M*^ was essentially independent of which 1.4 second interval of activity it was averaged over.

The main current source (positive transmembrane current, i.e., net positive charge leaving the neurons) was found in the soma subvolume (*n* = 3). The main current sinks (negative transmembrane current, i.e., net positive charge entering the neurons) were found in subvolumes containing proximal apical dendrites (*n* = 5, 6) and distal, branching apical dendrites (*n* = 12, 13, 14). We note that the transmembrane current profile summed to zero across depth, meaning that the sinks and sources balanced each others out (no neuron can be a net current sink nor source).

Of course, the neurodynamics and source/sink configurations seen in Figs 2 and 3 depended in a complex way on the particular neuronal morphology and the subcellular distribution of membrane mechanisms and synapses used in the simulations. The main objective of this work was, however, not to analyze these dependencies, but rather to explore how the ECS potential surrounding the neuronal population depended on whether diffusion was included in the simulations of the ECS dynamics. We investigated this for the particular scenario summarized in Figs 2 and 3, which was used in all simulations shown in the following, but with 84 seconds of simulated neurodynamics, and not only the seven seconds depicted in the figures.

We note that the neuron model by Hay et al. exhibited a rich repertoire of firing properties, including the occasional dendritic Ca^2+^ spikes seen in Fig 2B3. We refer to the original work for further details on the model properties [62]. In the following, the focus will be on how the simulated ECS potential (surrounding this given system) depend on whether ECS diffusion is accounted for.

### Diffusion does not affect the fast dynamics of the extracellular potential

Knowing the neuronal output to each ECS subvolume, we used the KNP-formalism to compute the resulting dynamics of ionic concentrations and the electrical potential in the ECS. Typically, ECS potentials are thought to mainly originate from various transmembrane current sources [16, 17]. Here, we explored whether diffusive currents in the ECS could constitute an additional source.

**Fig 4:**
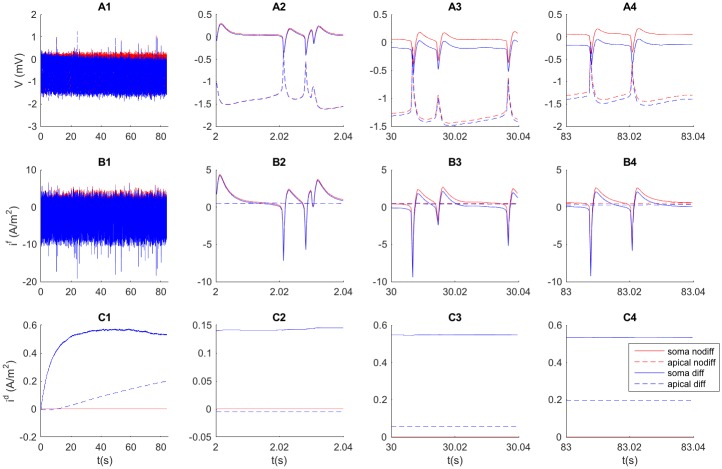
Ion dynamics on shorter time scales. (**A**) Time development of the ECS potential in the subvolumes containing the somata (*n* = 3, solid lines), and apical dendrites (*n* = 13, dashed lines). Time development of the ECS field current (**B**) and diffusive current (**C**) in the positive *z*-direction out from the soma subvolume (i.e., between *n* = 3 and *n* = 4, solid lines), and out from the apical dendrite subvolume (i.e., between *n* = 13 and *n* = 14, dashed lines). The first column (A1–C1) shows the signal for the entire 84 second simulation, while the three other columns of panels show the signal in three selected, brief intervals during the simulations. Red lines show the signal obtained when diffusion was assumed to be zero, while blue lines show the signal obtained with the full electrodiffusive formalism. Field currents varied at the same time scale as *V* (∼ milliseconds), while diffusive currents varied very slowly (∼seconds).

Fig 4A1–4A4 illustrates the dynamics of the ECS potential in two selected subvolumes (soma, *n* = 3, solid line; apical dendrite, *n* = 13, dashed line) due to the neuronal activity shown in Fig 2. Similarly, Fig 4B and 4C show the field currents and diffusive currents (respectively) from subvolume *n* = 3 to *n* = 4 (solid line) and from subvolume *n* = 13 to *n* = 14 (dashed line). For simplicity, we in the following discussion refer to the current from *n* = 3 to *n* = 4 as the current *out from the soma subvolume*, and the current from *n* = 13 to *n* = 14 as the current *out from the apical dendrite subvolume*. The first column (1) of Fig 4 shows the time course of these variables over the full simulation, while the remaining columns (2–4) show the time course over selected, shorter (40 ms) time intervals, which include only a few neuronal APs. Red curves represent the scenario without diffusion in the ECS simulations, while blue curves represent the scenario with ECS diffusion included.

When we explore the extracellular AP signatures (panels A2–4), we see that they had the same time course as the field currents (panels B2–4), while diffusive currents varied little at this fast time scale (panels C2–4). Diffusive currents thus had no impact on the fast temporal dynamics, and the AP signatures resembled those previously studied in models based on volume-conductor theory, where diffusive currents are neglected [34].

Somatic AP generation was due to an inward (depolarizing) current into the neuron followed by an outward (repolarizing current). Since the sum of transmembrane currents over the neuron as a whole (all ionic + capacitive currents) must be zero at all times, the dendritic branches experienced the opposite current configuration during the APs (outward currents followed by inward currents). Therefore, AP signatures in the apical ECS subvolume (dashed lines in Fig 4A2–4A4) had the opposite temporal profiles compared to what we observed in the soma subvolume (solid lines in panels A2–4).

Although the AP signatures were of the same order of magnitude in the soma and apical subvolumes, ECS field currents out of the soma subvolume were generally much larger than field currents between neighboring dendritic subvolumes (panels B2–4). The explanation lies in the spatial distribution of transmembrane inward and outward currents, and the rather unique role played by the soma. For example, a local inward current to the soma returned to the ECS in a widespread manner, i.e., it was distributed over the entire dendritic tree. Neighboring dendritic subvolumes therefore had similar AP signatures, implying that the ECS voltage differences (and therefore the field currents) between them were small.

The diffusive currents varied at a much slower time scale compared to field currents (Fig 4C). This was due to the slow time scale at which ion concentrations varied (as we shall explore further below). The diffusive current out of the soma region reached a peak value after around 30 seconds, after which it decreased slowly. The concentration build-up was slower in the subvolumes containing apical dendrites, and diffusive currents were smaller there, and still increasing at the end of the simulation (panel C1).

In the early part of the simulation, when diffusive currents were small, the ECS potential *V* was close to identical in the cases with and without diffusion (panel A2). However, as diffusive currents built up, they did have an effect on *V*, which was shifted to more negative values in the simulation with diffusion included compared to case without ECS diffusion (panel A3–A4). Towards the end of the simulation, diffusion had shifted *V* by about -0.2 mV in the soma subvolume. In the following, we shall explore this process in further detail.

### Diffusion depends on extracellular ion-concentration dynamics

**Fig 5:**
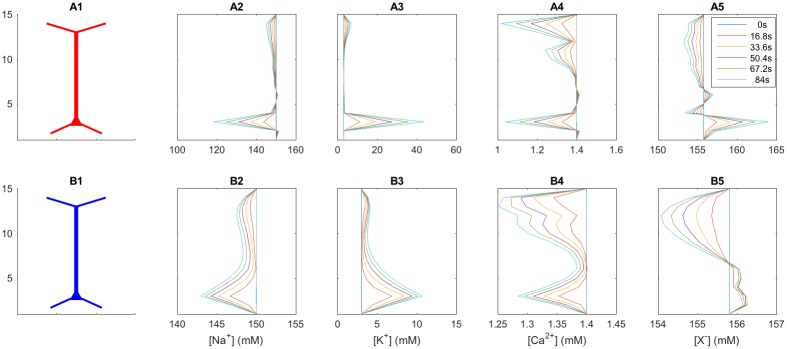
Extracellular ion-concentration profiles at selected time points. Spatial profiles of the ECS ion concentrations over the depth of the piece of tissue at selected time points. Deviances from baseline concentrations (*t* = 0) increase throughout the 84 second simulation. Simulations shown for the case with diffusion set to zero (**A**) and with diffusion included (**B**).

Diffusive currents in the ECS are proportional to concentration gradients in the ECS. To gain insight in the slow dynamics of the diffusive currents, we must therefore investigate the ECS ion-concentration dynamics. In our simulations, ECS concentrations varied due to ionic output from the neurons. Fig 5 shows how the ECS concentration varied over the tissue depth at selected time points. The deviations from the initial concentrations became gradually larger throughout the 84 second simulation, illustrating the slow time scale of ion-concentration dynamics in the ECS.

When diffusion was not included in the ECS simulations (Fig 5A), ionic transports were solely due to electrical migration, and were not biased towards following concentration gradients of distinct species. In this case, the ECS concentration profiles predominantly reflected the distribution of neuronal sources. For example, somatic AP generation caused a sharp decrease in the Na^+^ concentration and a corresponding increase in the K^+^ concentration in the soma subvolume, while the Na^+^ and K^+^ concentration changes were relatively small outside this subvolume (Fig 5A2 and 5A3). We note that the ion-concentration changes in the soma subvolume were unphysiologically high in the no-diffusion case. However, this was of no concern in the current study, since ion concentrations had negligible impact on the ECS dynamics in the case where diffusion was not included. (In this case the only effect on the ECS potentials came from the concentration dependence of the ECS conductivity, see Methods, Eq 11. However, for the present case the conductivity changes were found to be too small to have a visible impact on *V* in the simulations, see Discussion).

With diffusion included in the ECS simulation, the ion-concentration gradients across the depth of the piece of tissue became smoother (Fig 5B). For example, a fraction of the K^+^ expelled during somatic AP firing diffused out of the soma subvolume, and distributed across the entire tissue volume. In this case, the K^+^ concentration in the soma subvolume increased from a baseline level of 3 mM to slightly above 10 mM during the 84 second simulation, accompanied by a similar reduction in the Na^+^ concentration. These concentration shifts were within the range that can be expected under non-pathological physiological conditions (for K^+^, the limiting concentration between non-pathological and pathological conditions is typically estimated to be between 10 and 12 mM [7]).

The buildup of ECS concentration gradients explains the temporal development of the diffusive current that we observed in Fig 4C1. Early in the simulation, the diffusive current out of the soma subvolume (i.e., from *n* = 3 to *n* = 4) increased in an approximately linear fashion with time. This was because the local ion concentration in the soma subvolume (*n* = 3) increased in an approximately linear fashion due to the high neuronal output/input in this subvolume. As the ion-concentration gradients built up, diffusion from *n* = 3 to *n* = 4 increased, and the concentration increase in *n* = 3 became sublinear. Eventually, diffusion tended to smoothen out the ECS ion-concentration gradients (Fig 5A), and after about 30 s, diffusion between *n* = 3 and *n* = 4 experienced a slight decrease. A similar process took place over the entire tissue depth, but was slower further away from the soma, as the transmembrane ionic exchange was smaller there. In the apical dendrites (i.e., diffusion from *n* = 13 to *n* = 14), the diffusive current still increased in a close to linear fashion at the end of the 84 second simulation (Fig 4C1).

### Diffusive currents induce slow shifts in extracellular potentials

**Fig 6:**
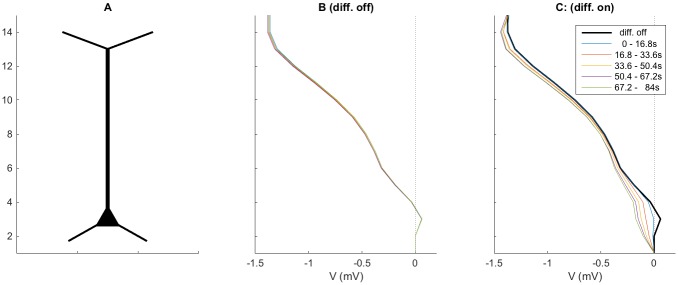
Extracellular potential profile with and without extracellular diffusion. **(A)** Tissue subdivided into 15 sub-volumes. **(B-C)** Distribution of electrical potential *V* over the depth of the piece of tissue for the situation where diffusion was assumed to be zero **(B)**, and for the situation with diffusion included **(B)**. The variables were low-pass filtered by taking the temporal average over the time intervals indicated in the legend. To facilitate direct comparison, the (constant) *V*-profile for the case without diffusion was also plotted in **(C)**.

Due to the slow nature of diffusive currents, we proceeded to investigate the slow time scale dynamics of the ECS potential. To do this, we took the time series of *V* (plotted for selected subvolumes in Fig 4), and split it up in five equal time intervals of 16.8 second duration (adding up to the total simulation time of 84 seconds). Next, we took the temporal average of *V* in these five intervals and obtained a (very) low-pass filtered version of the ECS potential. The results are displayed in Fig 6 showing how the low-pass filtered *V* was distributed across the tissue depth in the cases without (Fig 6A2) and with (Fig 6B2) diffusion included in the ECS simulations.

We first investigate the ECS voltage gradients obtained in the case where ECS diffusion was not included in the simulations (Fig 6B). In this case, there was an ECS voltage drop (of about 1.3 mV) from the soma subvolume to the subvolumes containing the apical dendrites. The drop in *V* was consistent with the neuronal source/sinks configurations that we observed earlier (Fig 3): Since the main neuronal current source (transmembrane current entering the ECS) was found in the soma subvolume (*n* = 3), while the sinks (transmembrane current leaving the ECS) were located higher up along the apical dendrites, there had to be an ECS current in the positive *z*-direction (corresponding to a negative voltage gradient in this direction) to close the current loop between the sources and sinks. Similar *V* profiles have been seen experimentally where sustained voltage profiles which vary by a up to several mV at spatial scales of millimeters have been seen in cortex [1, 3], hippocampus [26] and in the spinal cord [23].

We also note that the neuronal current sources/sinks were effectively constant at this slow time scale (Fig 3), meaning that they were essentially the same in all the five different time intervals in Fig 6. We would then *a priori* expect the ECS current to be constant over time as well. Without extracellular diffusion, this would in turn imply that also the ECS voltage gradient should remain constant throughout the simulation, which is indeed what is observed in Fig 6A2 (lines are on top of each others).

With diffusion included in the ECS simulations, the situation became more complex (Fig 6C). The gross features of the ECS voltage gradient resembled what we saw in Fig 6B. The similarity was not surprising, since the neuronal sources were identical in the two cases. However, with diffusion included, the ECS potential gradients no longer remained constant throughout the simulation (Fig 6C). The time-dependent variations were most pronounced in the soma subvolume where the ECS potential decreased by about 0.2 mV over the time course of the simulation. This shift in *V* was caused by diffusive currents along the ion-concentration gradients that built up during the simulation, and was the same shift that we previously observed in Fig 4A4. A detailed physical interpretation of the diffusion-induced shifts in the ECS potential is provided in the following subsection.

### Diffusive effects on extracellular potentials explained by Kirchhoff´s current law

**Fig 7:**
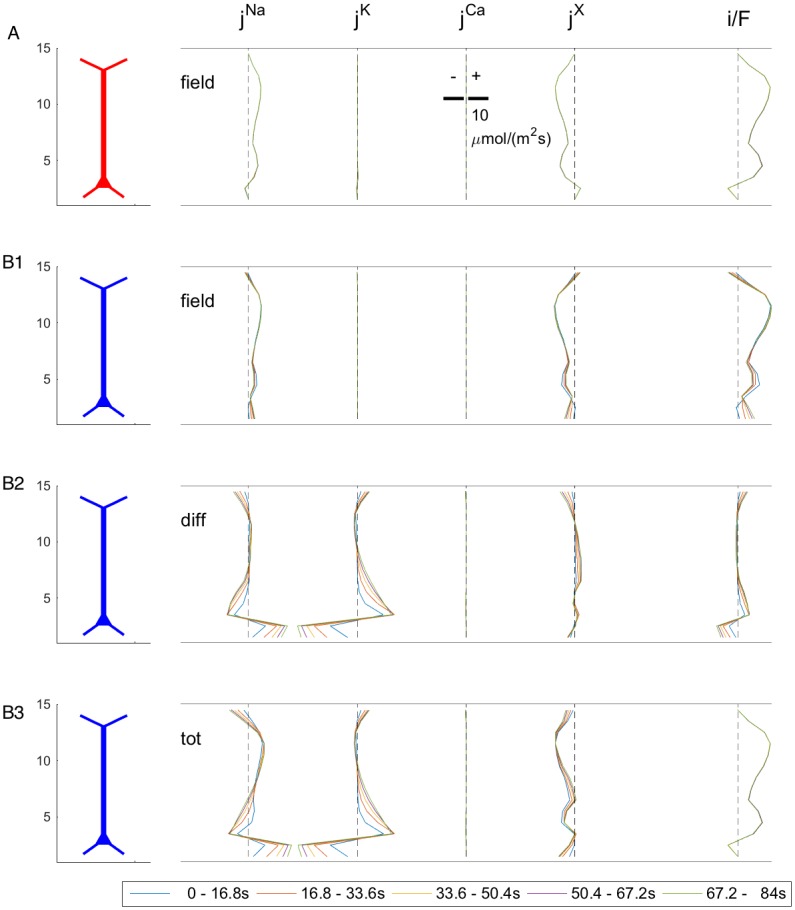
Extracellular flux densities of ions and net charge. Time-averaged extracellular flux densities in the cases without (**A**) and with (**B**) extracellular diffusion. In the latter case, the total flux density **(B3)** was subdivided into the field-driven **(B1)** and the diffusive **(B2)** component. When the curves are to the right/left of the dashed vertical lines, they represent fluxes in the positive/negative *z*-direction, respectively. The flux densities were computed as the temporal mean over time intervals indicated in the legend. The scale bar was the same for all flux densities, including the electrical current density (rightmost column), which was given in units of the unit charge: *i*/*F* = *j*^*K*+^ + *j*^*Na*+^ + 2*j*^*Ca*2+^ − *j*^*X*−^.

To obtain a more thorough understanding of the interplay between the potential *V* and diffusive currents, we next plotted the ECS fluxes of all ion species (K^+^, Na^+^, Ca^2+^, and X^-^) in the cases without and with extracellular diffusion (Fig 7). Also here, the focus was on the long time-scale dynamics, and we compared the time-averaged fluxes taken over five 16.8 second time intervals (same procedure as used for *V* in Fig 6). In the rightmost column in Fig 7, we have also plotted the total electrical ECS current associated with the ionic fluxes (the definition is given in the caption of Fig 7).

When ECS diffusion was not included in the simulations, all ion transport in the ECS were due to the electrical field (Fig 7A). In that case, most of the transports were mediated by the most abundant ion species in the ECS, which in our simulation were Na^+^ and X^-^. Due to the negative potential gradient between the subvolumes containing the soma and apical dendrites (Fig 6C), the positively charged Na^+^ ions were driven away from the soma subvolume, while the negatively charged X^-^ ions were driven towards the soma subvolume. Both these ion fluxes amounted to a net electrical current away from the soma subvolume, i.e., a positive current in subvolumes above the somata (*n* > 3) and a negative current in subvolumes below the somata (*n* < 3).

In simulations including extracellular diffusion we plotted the ECS flux densities due the electrical field (*j*^*f*^) and diffusion (*j*^*d*^) separately (Fig 7B1 and 7B2), as well the total flux density (*j*^*f*^ + *j*^*d*^, Fig 7B3). As AP firing evoked a decrease/increase of Na^+^/K^+^ in the soma subvolume, ECS diffusion drove Na^+^ into this subvolume, while it drove K^+^ out of this subvolume (Fig 7B2). As these two cation fluxes were oppositely directed, the net diffusive charge transport (*i*^*d*^/*F*) was smaller than the charge transported by Na^+^ and K^+^ separately. However, the diffusive fluxes still gave rise to a net electrical transport of the same order of magnitude as the field-driven current, especially around the soma subvolume (compare current densities in panels B1 and B2 in Fig 7).

The ionic fluxes in the ECS differed quite significantly between the cases with and without ECS diffusion (compare flux densities in panels A with B3 in Fig 7). However, the net electrical current in the system were identical in the two cases (compare current densities in panels A and B3). This can be understood from basic electric circuit theory: As the neuronal transmembrane sources/sinks were identical in the two cases, the same had to hold for the net extracellular current. Otherwise, the current loop would not be completed. This leads to the following key insight: Since the net electrical current density (*i*^*tot*^ = *i*^*f*^ + *i*^*d*^) was independent of whether diffusion was present in the model or not, an increase in *i*^*d*^ had to be accompanied by a corresponding decrease in *i*^*f*^, and vice versa. A time-dependent variation of diffusive currents therefore by necessity evoked a time-dependent variation of the field currents (Fig 7B1)). As *i*^*f*^ was proportional to the voltage gradient, this in turn implied that the ECS voltage gradients varied with time, as observed in Fig 6.

### Diffusive currents change the power spectra of local field potentials

So far, we have demonstrated that diffusive currents can have quite substantial effects on ECS potentials, at least on a slow time scale. As a next inquiry, we would like to know the frequency range in which diffusion can be expected to have an effect on recorded ECS potentials, and in particular whether diffusion can be expected to affect experimental LFP recordings where the low-frequency cut-off typically ranges from 0.1 Hz to 1 Hz (see e.g., [29, 30]).

**Fig 8:**
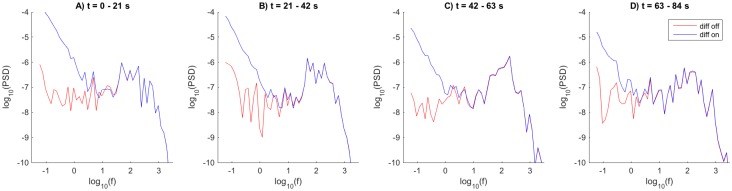
Effects of diffusion on power spectral densities (PSDs) for the ECS potential in the soma subvolume. (**A–D**) show the power spectra of *V* in the soma subvolume (*n* = 3) under four consecutive 21-second time intervals of the 84 second simulation. Units for frequency and PSD are Hz and mV^2^/Hz, respectively.

We limited this study to ECS potentials recorded in the soma subvolume, where the diffusive effects were most pronounced in our model. Fig 8 shows the power spectral densities (PSDs) of the ECS potential recorded outside the somata (*n* = 3), where *V* was obtained as in the above simulations in Figs 4–7). To explore the development of the PSDs over the time course of our simulation, we split the 84 second time series of *V* into four 21 second intervals, and computed the PSD for these time intervals separately.

A first observation is that the PSDs for the simulations without (red lines) and with (blue lines) ECS diffusion differed dramatically for the lowest frequencies, where the presence of diffusion boosted the PSD by up to several orders of magnitude. Contrarily, for the highest frequency components the PSDs were close to identical in the cases without and with diffusion (red and blue lines overlap). This was as expected from our previous analysis where we saw that diffusion was important for the slow, but not the fast system dynamics (Fig 4). The cross-over frequency for which the diffusion contributed negligibly to the PSD, was for all four time intervals depicted in Fig 8 seen to be in the frequency range between 1 and 10 Hz. Extracellular diffusion was thus found to have effect on the PSD for frequency components well within the range typically considered in recordings of LFPs *in vivo* [29, 30].

The PSDs obtained with no ECS diffusion (red lines) were quite constant throughout the simulation, while the PSDs obtained with ECS diffusion included (blue lines) were generally higher for the earliest time intervals (compare panels A and D). To provide a hand-waving explanation to the latter, we start by noting that the contribution of diffusion to the local PSD essentially depended on the absolute value of the temporal variation of local ion concentration (i.e., on $|{\dot{c}}^{k}|$, see S1 Appendix), which in turn depended on two competing processes.

The first process was the local neuronal output of ion species *k*, which was roughly constant at the long timescale considered here. The second process was ECS transportation of ion species *k* out from/into the local region. Generally, these two processes had opposing effects on the local ion-concentration dynamics (i.e., when neurons expelled K^+^ into a given subvolume, ECS transports tended to drive K^+^ out from that subvolume). Early in the simulation, ECS concentration gradients (and thus ECS diffusive transports) were small, and the time development of the local concentration was approximately proportional to the neuronal output. At a later stage, ECS concentration gradients had built up, and the competing diffusive process had increased. Then local concentrations changed more slowly with time.

### Diffusion can evoke extracellular potentials even in absence of neural current sources

In the rather complex scenario studied so far, transmembrane and extracellular currents interacted (as is, of course, the case in real brain tissue). However, diffusive fluxes and currents in the ECS can in principle exist even without on-going neuronal sources, provided that there are concentration gradients present in the ECS. To improve our understanding of diffusion-generated potentials, we explored them also in such a simplified scenario. For simplicity, we used the same simulation as above (Figs 2–7) to generate reasonable ECS concentration gradients needed in the simplified scenario. However, this time we turned off the neuronal current sources midways in the simulation (i.e., after 42 seconds), and analyzed the ECS dynamics in last 42 seconds of the simulation when the ECS dynamics was solely due to diffusion along the concentration gradients that had built up during the first 42 seconds of the simulation.

For this scenario, only the simulations *with* ECS diffusion included gave non-trivial results (when extracellular diffusion was *not* included, the ECS voltage gradient instantly turned to zero when the neuronal current sources were removed, and the extracellular ion fluxes immediately stopped). This can be easily understood from the current conservation laws upon which the KNP formalism was based, stating that the sum of currents into an ECS compartment should be zero (Fig 1C). In the simplified scenario, there were no transmembrane sources after 42 seconds, and with no diffusive currents between ECS subvolumes, the field currents (and thus voltage differences) between ECS subvolumes must by necessity also be zero.

**Fig 9:**
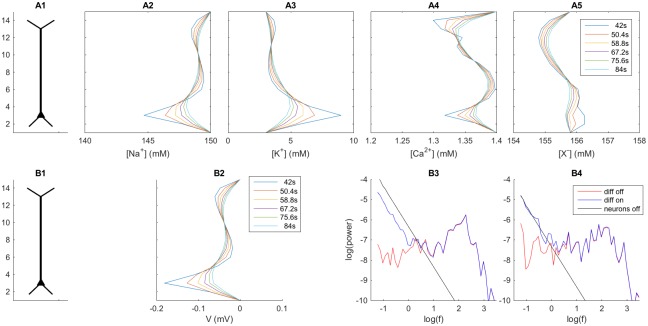
Extracellular dynamics without neuronal current sources. ECS dynamics in time interval between *t* = 42 s and *t* = 84 s after turned off (at *t* = 42 s). (**A2**-**A5**) Profiles of ECS ion concentrations at selected time points. The ion-concentration gradients alone gave rise to an electrical (diffusion generated) potential in the ECS. (**B2**) ECS profiles of the diffusion potential *V*. The depicted potential corresponds to the temporal averaged *V* taken over 8.4 second intervals indicated in the legend. (**B3**-**B4**) Power spectral density (PSDs) of the potential (*V*) in the soma subvolume (*n* = 3) due to ECS diffusion (black line) under two consecutive 21-second time intervals. For comparison, the PDSs of the original simulations (i.e, when neuronal sources were not turned off) were also plotted (red and blue lines). The legend in (A2) applies to all concentration profiles (A2–A5). The legend in (B3) applies to all PDSs (B3–B4). Units for frequency and power are Hz and mV^2^/Hz, respectively.

The simulations with ECS diffusion included are shown in Fig 9. Panels A2–5 show the ECS concentration profiles at selected time points *after* the neuronal sources were turned off at *t* = 42 s. Initially (i.e., at *t* = 42 s), ionic concentrations of Na^+^, K^+^, Ca^2+^ and X^-^ in the soma subvolume had been shifted by approximately -5.1 mM, 6.0 mM, -0.1 mM and 0.7 mM, respectively, relative to the baseline concentrations. We note that these shifts fulfilled the requirement of local electroneutrality, i.e., did not correspond to any net change in the local charge density: *Σ*_*k*_(*z*^*k*^*c*^*k*^) = (−5.1 + 6.0 − 2 × 0.1 − 0.7) mM = 0. Here *z*^*k*^ and *c*^*k*^ are the valence and concentration, respectively, of ion species *k*.

The deviations from baseline concentrations were smaller outside the soma subvolume, and the concentration gradients out of the soma subvolume were quite steep. Diffusive currents along these gradients gave rise to a diffusion potential, which at *t*=42 s peaked in the soma subvolume where *V* was about -0.17 mV (Fig 9B2). Diffusion-evoked voltage gradients like this are well understood, and have been observed in many systems with spatial variation in ion composition [3, 20–22].

With no neuronal sources present, the ECS concentration gradients were gradually smoothed over time (i.e., for *t*> 42 s). Consequently, the ECS voltage gradients decayed. At the end of the simulation (i.e., for *t* = 84 s), *V* was about -0.05 mV in the soma subvolume. The PSD corresponding to this decay process is depicted by the black lines in Fig 9B3 and 9B4. Since the concentration gradients became gradually smoother, the power was generally higher during the first 21 s after the neurons were turned off (Fig 9B3) than in the proceeding 21 s (Fig 9B4). In both cases, the PSDs were very close to a 1/*f*^2^ power law (the fitted power-law coefficients were 1.998 in panel B3 and 2.02 in B4). This so-called *Brownian-noise* power law essentially follows from an exponential decay of local ion concentrations, and can be derived analytically (see S1 Appendix).

For comparison, we also show the PSDs of the simulation with neuronal sources included (the red and blue lines in Fig 9B3 and 9B4 are the same as in Fig 8C and 8D, respectively). Also in the presence of neuronal sources, the electrodiffusive ECS process roughly followed a 1/*f*^2^ power law for low frequencies where diffusion dominated (blue line and black line close to parallel for *f* < 10 Hz).

Comparing the blue and black lines, we further note that the removal of neuronal current sources at *t* = 42 s increased the low-frequency components of *V*, especially during the first 21 second time interval after the time of the sources offset (Fig 9B3). To explain this, we may recall that the diffusive power spectrum is proportional to the absolute value of the temporal variation of local ion concentration ($|{\dot{c}}^{k}\left|\right)$. As argued above, this value depends on the balance between two competing processes, i.e., the local neuronal output of ion species *k* and the ECS transports of ion species *k* out from/into the local region. The observation in Fig 9B3 simply implies that the local concentration *approached* the baseline levels faster when the neuronal sources were turned off (black line) than it *diverged* from the baseline level in the case when the neuronal sources were kept on (blue line).

In reality, transmembrane current sources and ECS transport processes *do* interact, and the correct electrodiffusive PSD is predicted by the blue line in Fig 9B3 and 9B4. Likewise, the predicted maximum frequency that will be affected by diffusion is in the frequency range 1–10 Hz where the red and blue lines in Fig 9B3 and 9B4 merge. However, we still believe that the study of the simplified decay process process (with neuronal sources turned off) provide useful insights to how ECS diffusion can affect the PSD. Firstly, the simplified ‘decoupled’ model nicely illustrated that ECS diffusion gave rise to a 1/*f*^2^ contribution to the PSD, as we saw above. Secondly, we propose that the crossing point between the PSD obtained for the diffusive process alone (with concentration gradients representable for what one typically see in the system) and the PSD obtained from neurodynamics when diffusion was not included (black vs. red line in Fig 9B3) may serve as a crude estimate of the maximum frequencies for which diffusion can be expected to influence the PSD. For example, the crossing point between the red and black line in Fig 9B3 was found in the frequency range 1–10 Hz, which agreed with the frequency range where the blue and red lines merged. We will provide further arguments for the usefulness of the simplified scenario further in the Discussion. So far, we conclude that in the current model, diffusive processes affected ECS potentials for frequencies up to several hertz.

## Discussion

We tested the hypothesis that, unlike what has been assumed in previous theoretical analysis based on volume-conductor theory [18, 29, 30, 32, 33, 36], ECS potentials can be influenced by the presence of diffusive currents in the ECS. To explore this, we simulated the ECS transport of ions in a piece of neural tissue, stemming from the activity of a small population of ten pyramidal cells. We explored a scenario with large, but biologically realistic, fluctuations in ECS concentrations and compared simulations where diffusive currents were included in the ECS dynamics with simulations where diffusive currents were set to zero. The following key findings were made: (i) ECS diffusion shifted the local ECS potential by up to ∼0.2 mV. (ii) The diffusion-evoked potential shifts occurred at a slow time scale, and their contribution to the PSD of the ECS potential followed a 1/*f*^2^ power law at the lowest frequencies. (iii) In the model, the diffusive process had a non-negligible impact on the PSD for frequencies up to ten hertz, i.e., standard volume-conductor theory which ignores diffusion in the ECS, would estimate the PSD correctly only for frequencies higher than about ten hertz.

We note that effects that diffusion had in the simple, ten-neuron system considered here is likely to be larger than under most realistic conditons. This is partly because the concentration gradients in the model were in the upper range of what has been observed under non-pathological, experimental conditions, and partly because real tissue contains a multitude of additional mechanisms which could serve to reduce concentration gradients and at the same time boost the part of the PSD that reflects transmembrane current sources/sinks (see below for a more detailed discussion). In most scenarios, we expect many models that exclude diffusion still to give quite accurate results for the problems in question. However, we regard the current modelling study as a demonstration that, as a generality, diffusive currents can not be assumed to have a negligible impact on ECS potentials, whereas the actual role of diffusion must verified in each specific case.

### Kirchhoff-Nernst-Planck formalism

#### Relationship to other modelling schemes

What we here have coined the *KNP formalism* was developed in previous work where we derived a mathematical formalism for simulating buffering of extracellular K^+^ by astrocytes [28, 57]. A very similar formalism was developed (in parallel) in the heart-cell community in the context of a model of ischemia [58]. Our buffering model accounted for electrodiffusive processes in the intra- and extracellular domain, and was essentially an expansion of the previous model by Qian and Sejnowski [64], which only considered the intracellular domain.

The KNP formalism represents a simplification of the computationally expensive PNP-solvers, which derive the local potential from Poisson’s equation (e.g., [47–51]). The PNP system has been thoroughly analyzed in a series of previous works by Mori, who also proposed a series of simplified models and studied their validity under different conditions [53–56, 65]. The KNP formalism can be regarded as a simplified version of the electroneutral model proposed by Mori [52, 54, 65]. In the current application of the KNP model, we tailored the formalism to study transport processes in neuronal tissue at a relatively large spatiotemporal scale.

In the original application of the KNP formalism, we modelled both the intra- and extracellular space explicitly [28]. In the current application, we introduced a hybrid modelling framework, where the KNP formalism was only applied to the ECS, while the intracellular dynamics was computed with the NEURON simulator [59, 60]. The NEURON simulator is an efficient standard tool for computing the dynamics of morphologically detailed neurons. It can be combined with algorithms for handling the intracellular dynamics of selected ion species due to local transmembrane influxes/effluxes and decay processes. (In most neural models, such algorithms are typically an exception used only for the signalling molecule Ca^2+^, see e.g., [62, 66–68].) However, intracellular electrodiffusive processes are so far not an integral part of the NEURON simulator. A limitation with the hybrid scheme is therefore that electrodiffusive processes are only accounted for in the ECS domain, where the KNP-formalism is used. An important advantage with the hybrid scheme is that it lends itself to be used as a supplement to compute the ECS dynamics (of ion concentrations and the electrical potential) of the multitude of already available neural or neural network models based on the NEURON simulator (such as, e.g., the Blue Brain simulator [69]). Previous tools developed to compute extracellular potentials from NEURON based models such as LFPy [61] have not incorporated effects of ionic diffusion in ECS.

#### Electroneutrality assumption

Along with other electroneutral models [53–56, 58, 65], the KNP formalism provides a means of deriving the local potential *V* from the physical constraint that the (intra- and extracellular) bulk solution is electroneutral [28]. This approximation was used as early as in 1890 by Planck, who described electrodiffusion in electrolytes [70]. In the current application, this approximation means that any nonzero local charge density within the system is identified as a charge that sits on a capacitive membrane and uniquely determines the local transmembrane potential of an excitable cell. Put differently, the KNP scheme assures that the sum of ionic currents into a given tissue sub-volume equals the sum of capacitive (non-ionic) currents over the cellular membranes that populate the sub-volume (as illustrated in Fig 1C), so that no net charge is found in the bulk.

The assumption that bulk solutions is electroneutral is not strictly true, as has been the topic of many discussions (see e.g., [45, 71]). Indeed, Fig 9 showed that in the presence of diffusion, we could obtain a nonzero voltage gradient even in the absence of neuronal sources, an observation which is incompatible with the notion of a strictly electroneutral ECS. However, it has been shown that invoking the electroneutrality assumption is equivalent to invoking the limit of the exact PNP treatment when charge-density-dependent effects become small [71], and that the electroneutral model works as an excellent approximation at spatiotemporal scales larger than microseconds and micrometers [52, 65].

#### Diffusion potentials

Diffusion-generated potentials are well known in electrolyte theory. Often they are referred to as *liquid junction potentials*, since they are most pronounced at the boundary between two solutions of different ion composition [20–22, 72].

In reality, the genesis of liquid junction potentials is a three-step process that requires (i) initial ion-concentration gradients that are such that diffusion will drive a net electrical charge in some direction, (ii) a charge separation associated with the diffusive process, and (iii) an electrical potential that arises from the charge-separation process, and opposes further charge separation. This diffusion-generated potential (iii) represents a quasi steady-state scenario where electrical drift and diffusive drift are opposing and in equilibrium. Simplified equations for computing diffusion potentials include the Henderson equation and the Goldman-Hodgkin-Katz equation (see e.g., [22], and S2 Appendix). The relaxation towards this quasi-steady state occurs very rapidly, i.e., on the nanosecond timescale [73]. Furthermore, the number of ions that constitute the net charge density during equilibrium is about nine orders of magnitudes smaller than the number of ions present [72]. The KNP formalism bypasses the rapid equilibration process by assuming that the quasi-steady state is reached instantaneously, and derives the value for *V* associated with the equilibrium state. In doing so, the KNP formalism implicitly neglects the tiny local charge separation associated with the charge relaxation process.

To get an intuitive understanding of the diffusion potential, it may help to compare it with the (in neuroscience) more familiar cellular resting potential, which is typically computed from the Goldman-Hodgkin-Katz (GHK) equation (see S2 Appendix). The GHK-equation predicts the equilibrium potential between two compartments *A* and *B* with different ion compositions, i.e., the potential difference at which the diffusive current from *A* to *B* equals the field driven current from *B* to *A*. When the resting potential is computed, the compartments *A* and *B* represent the inside and outside of an excitable cell membrane. However, if we let *A* denote the subvolume *n* = 1 where the ion concentrations (by constraint) had the baseline values (Table 1), and let *B* denote the compartment *n* = 3 with concentrations as in Fig 9 at *t* = 42 s, the GHK equation (see S2 Appendix) predicts a potential difference of −0.17 mV between the two compartments. This agrees with the value we got in Fig 9 when neuronal sources were turned off at *t* = 42 s.

#### Extracellular conductivity

Unlike in volume-conductor theory, where a fixed value typically is used for the ECS conductivity [18, 32–34], the KNP formalism models the conductivity (*σ*) as a function of the number of free ionic charge carriers, weighted by their mobility and valence (cf., Eq 11). In neural tissue, the main charge carriers are typically believed to be K^+^, Na^+^, and Cl^-^. The model included K^+^, Na^+^, Ca^2+^ and an unspecified anion species (X^-^). The latter essentially represented Cl^-^ in the biological system and was given the same baseline ECS concentration and diffusion constant as Cl^-^. The model thus included the main charge carriers, and with the initial ion concentrations that we used, we obtained an ECS conductivity (*σ* = 0.76 S/m).

In the literature, there are quite some variations in values that are given for the ECS conductivity, and also variations in how this quantity is defined. In the current study, we used the porous medium approximation [74], and explicitly accounted for the fact that ECS currents only go through a volume fraction of about 0.2 of the tissue volume (see methods). However, it is common to rather define an *apparent* tissue conductivity, *σ*′, which is defined using the tissue as a whole as reference volume for ECS currents [4]. In our case, the apparent conductivity was thus *σ*′ = *ασ* ≃ 0.15 S/m. For comparison, Chen and Nicholson found an apparent conductivity of *σ*′ = 0.1 S/m [4], while other computational studies of local field potentials and current-source densities have used values *σ*′ ∼ 0.3 S/m [18, 32–35, 75]. Our estimate thus lies between the previously estimated values for *σ*′, and is relatively close to the value used by Chen and Nicholson [4].

Relative variations in ion concentration were quite small in the simulations studied here (at least when it comes to the most abundant species). In addition, such variations tended to be asymmetric (e.g., decreases in K^+^ were accompanied by increases in Na^+^), meaning that variations in the net number of free charge carriers were even smaller than variations in individual ion species. Therefore, *σ* only varied by a few percent relative to the initial value during simulations. As verified in additional test simulations where *σ* was pegged at the initial values, these variations had no significant effect on the simulation results.

Albeit its concentration-dependent magnitude, the conductivity (as defined here) was essentially a pure, resistive conductor, i.e., it was independent of the frequency of currents passing through it. A frequency independent conductivity finds support in recent experiments [76] (although there are also experiments that have indicated otherwise [77]).

### Model assumptions

The simplified model set-up used here have several limitations. Firstly, *V* was computed as an averaged value over a large ECS volume, and comparison between this and experimental recordings of *V* with point-like electrodes with small contacts is not straightforward. Secondly, brain tissue contains many types of neurons, which are distributed with somata in different depth layers (see, e.g., [18, 69, 78]), whereas we only included one. Thirdly, we did not include synaptic connections between neurons. Such connections could induce a level of synchrony in the neuronal firing, which likely would influence the power spectrum of the ECS potential [16, 35]. Fourthly, we assumed that spatial variations in the electric potential and ion concentrations occurred only in one spatial dimension. This is clearly not strictly true, and some aspects of the estimated power spectra are likely to depend on the three-dimensional nature of the real system. Fifthly, the presently used multicompartmental neuronal model [62] (together with most other available multicompartmental models) does not include ionic uptake mechanisms such as Na^+^/K^+^-pumps. Such mechanisms, along with glial uptake mechanisms [28, 79], would generally act to maintain the ECS ion concentrations closer to the baseline levels than what we predicted with our model. These shortcomings are discussed in further detail below.

#### No feedback from extracellular space to neurons

To compute the ion-concentration dynamics in the ECS (Fig 5), we counted the number of ions exchanged between the neurons and the ECS in simulations of the multicompartmental neural model [62]. For simplicity, we assumed that that there was no feedback from the ECS dynamics to the neurons. That is, we did not account for changes in neural reversal potentials due to changes in ECS ion concentrations [9, 12], or ephaptic effects of ECS potentials on neuronal membrane potentials [53, 80–82]. Such feedback mechanisms would likely influence the neurodynamics. However, we do not believe this to be a main concern with the current study, as the main focus was not the neurodynamics as such, but rather the ECS dynamics resulting from it. Having no feedback also gave us the advantage that we could have exactly the same neurodynamics when comparing the ECS dynamics in the cases without or with diffusion in the ECS.

#### Unspecific ion species

Only a subset of the transmembrane currents in the multicompartmental neuron model [62] were ion specific, and we therefore assumed that all non-specific currents were mediated by an unspecified anion species (X^-^). For simplicity, we assumed that all non-specific currents (including the leakage current, synaptic currents, and the currents through non-specific, active ion channels were mediated by the same ion species X^-^.

Although this is an inaccurate assumption (e.g., leakage currents are composed of several ion species, and not only one, and the ion channel *I*_*h*_ in the neural model [62] is in reality a cation-channel), it was not critical for the simulation outcome. One reason for this is that the ECS ion concentrations did not have any significant effects on the ECS conductivity (as clarified above), so that the field currents depended little on the composition of ions in the ECS. As for the diffusive currents, the concentration gradients were most dramatic for K^+^ and Na^+^, and the remaining ion species (X^-^ and Ca^2+^) gave only minor contributions to ECS diffusion. A subdivision of X^-^ into different ionic species would therefore expectedly not change our qualitative findings.

#### Simplified neuronal model system

Albeit internally consistent, the model system represented a crude simplification of the complexity of real tissue, where an intricate circuitry of many different neuron species are likely to contribute to the ECS dynamics. The small population of 10 pyramidal cells used in the current study will likely create a bias towards strong concentration gradients surrounding the soma subvolume (*n* = 3 in Fig 1). In addition, brain tissue contains neuronal [83] and glial [2, 3, 6] uptake mechanisms (in particular Na^+^/K^+^-exchangers), which were not included in the current model. Such uptake mechanisms work to keep ECS concentrations close to baseline levels, and significant changes in ECS ion concentrations are therefore likely to occur only in cases when the neuronal activity level is too intense for such clearance mechanisms to keep up. The ionic concentration gradients predicted in Fig 5 are therefore likely to be an overestimation of the ion-concentration gradients that would realistically build up during the relatively moderate AP firing activity of the small neuronal population considered here. For comments on how non-included cellular mechanisms could influence the main findings in this work, we point to the discussion below on effects of ECS diffusion currents on measured PSDs.

#### Volume-averaged potential

Recorded ECS potentials depend on the distances between the recording electrode and the neuronal current sources [31]. For example, ECS signatures of APs are only large in the vicinity of the neural membrane, while slower signals can have a longer spatial reach [33, 35]. A direct comparison between *V* as determined by the formalism presented here (averaged over a ECS subvolume), and *V* measured by point electrodes, would require a generalization of the model to three spatial dimension, using a relatively fine spatial resolution. However, also in the present implementation, *V* was determined from current conservation laws, and followed the same time course as (Fig 4) the ECS signals seen in previous studies [33, 35]. In addition, the estimates of *V* in the current work showed sustained ECS profiles (Fig 5) that were qualitatively similar to those observed experimentally [1, 3]. We thus believe that the large scale (volume averaged) *V* considered in the current study represents a useful quantity for assessing relative contributions of field currents and diffusive currents at a tissue level.

#### Correlation effects introduced with population size and geometry

Two important model assumptions could influence the scaling of the PSD observed in this study: the grouping of *N* neurons into a joint population output *I*^*M*^ into each ECS subvolume, and the assumption of a 1D system geometry.

Regarding the population size, the *N* neurons in the current model received uncorrelated synaptic input, but with the same (time-averaged) input statistics. This means that the *N* neurons produced output where the fast components were uncorrelated (e.g., APs were unique for individual neurons) and the slow components were correlated (e.g., the time averaged output was the same for all neurons). Thus, we would expect the high-frequency part of *I*^*M*^ to sum as uncorrelated noise (the amplitude scales roughly like $\sqrt{N}$), and the low-frequency part to sum as correlated signals (the amplitude scales roughly like *N*). That is, the PSD would be dependent on the system size, so that an up-scaling of the system (increasing population size *N* and ECS volume by the same factor) would penalize the high-frequency part of the PSD relative to the low-frequency part. Such population effects have been demonstrated in a previous study, where the high frequency part of the ECS potential was found to scale sublinearly with the number of APs elicited in a volume [33].

Similarly, correlation-related effects could also be introduced with the 1D-assumption. In a hypothetical 3D model, the population in Fig 1 would be surrounded by similar populations. In the 1D model, the ECS currents in the lateral directions were by construction zero, which would be equivalent to having zero gradients of the voltage and concentration in the lateral directions. In a (hypothetical) 3D model, this would only occur in the case when neighboring populations were perfectly correlated, which is then an implicit assumption in the 1D model. This could be a good assumption for slow frequency components, but not for the fast components (i.e., the neighboring populations could share input statistics, but not exact AP-spike times). Hence, a transition to a 3D model would likely also penalize the high-frequency part of the PSD relative to the low-frequency part.

Diffusive currents were mainly found to influence the slow components of the PSD, and it is unclear whether the key findings regarding these would be influenced by choice of population size and model dimensionality. This would, however, be a natural topic for future investigations (see below).

### Model predictions

In the current section we discuss the predictions that we made regarding diffusion-generated electric potentials, and to which degree these can be expected to reflect realistic experimental scenarios. To clarify the discussion, we start by labeling the three situations that we have studied P1, P2, and P3, respectively. We refer to Fig 9B3 and 9B4, where all three situations (P1–P3) are represented. The blue line (P1) represents ECS dynamics surrounding an active neuronal population in the realistic scenario described by the full electrodiffusive formalism. The red line (P2) represents the situation where ECS diffusion was neglected so that the ECS potential was given exclusively by the distribution of transmembrane sources (cf., standard volume-conductor theory [32]). Finally, the black line (P3) represents the situation where the neuronal sources had been turned off, so that the ECS potential was driven exclusively by concentration gradients in the ECS. The concentration gradients could in principle be imposed as an initial condition in the system, independent of the neural model, meaning that P3 and P2 were essentially independent processes. As we shall see below, this independence is useful for analyzing our results, and for comparing them to previous studies.

#### Magnitude of diffusion-generated potential (P3)

Figs 6 and 9 showed that the diffusion-generated potential shifts in the ECS developed on a slow time scale. Slowly varying ECS potentials have also been reported in several experimental studies, and may be of the order of several millivolts [1, 3, 13, 23–26]. Generally, the main source of these slow potentials is not believed to be ECS diffusion, but rather glial buffering currents triggered by increases in ECS K^+^ concentrations [3], i.e., on transmembrane current sources that were not included in the computational model studied here. However, based on recorded concentrations differences between different cortical layers during neuronal hyperactivity, Dietzel et al. [3] estimated (using the Henderson equation [84]) that ECS diffusion could contribute to such shifts by maximally 0.4 mV, a finding that they also verified experimentally in a simplified setup. These estimates depended solely on differences in ion-concentration compositions between different cortical regions, and could thus be compared to our scenario P3. For the concentration gradients built up at the time when the neuronal currents were turned off (*t* = 42 s), we predicted a diffusion generated potential of about 0.17 mV (Fig 9). This was smaller than, but of the same order of magnitude as the maximal shifts estimated in [3].

The magnitude of diffusion potentials depends on spatial variations of ion concentrations, and may be large in non-biological systems (see e.g., [85]). In brain tissue, however, concentration differences are likely more moderate, and diffusion potentials larger than a few tens of a millivolt will probably be rare.

#### Power spectrum of diffusion-generated potential (P3)

While the magnitude of slow diffusion potentials finds support in previous experimental studies, no previous study has to our knowledge systematically investigated the PSD associated with their temporal development. In the current study we found that diffusion could have an effect on the PSD for frequencies as high as ∼10 Hz. In the discussion following Fig 9B3, we suggested that we could approximate the frequency range where diffusion had an effect with the frequency range where the PDS of the diffusive process alone (P3) had a magnitude that was similar to, or higher than, the PSD predicted from the transmembrane sources alone using volume-conductor theory (P2). We note that this is only an approximation, i.e., without neuronal sources as in process P3, the ion-concentration dynamics and thus ECS diffusion were not identical to that in the full model (P1). The approximation was useful as it allowed us to compare two independent processes (P2 and P3). Whether the predicted frequency range was realistic, can then be boiled down to a question regarding the realism of the PSDs obtained separately for the neuronal model (P2) and with pure ECS diffusion (P3).

The analysis of P3 led to the clear observation that the (undisturbed) diffusion potential followed a 1/*f*^2^ power law, as we also predicted analytically (see S1 Appendix). The realism of the diffusion-generated ECS potentials (P3) depend predominantly on whether the ion-concentration gradients as such were physiologically realistic, i.e., independently of which underlying neuronal process gave rise to them. Regarding ECS concentrations, most experimental data are available for K^+^, and the simulated K^+^ concentrations were within the range reported experimentally in different systems [1, 3, 4, 6, 8, 24, 86, 87]. For example, the simulated K^+^ gradients in Fig 5B bear some resemblance to K^+^ concentration gradients seen in the experiments by Cordingley and Somjen, where the K^+^ concentration varied with about 4 mM over the depth of cortex (see Fig. 5 in [1]). In line with previous estimates, the simulated shifts in the concentrations of other included ion species were smaller or of the same order of magnitude as for K^+^ [3, 88], and should also be physiologically realistic.

As for the temporal aspect, the simulated ion-concentration variations occurred at a timescale of tens of seconds, which is also similar to what has been seen in experiments (see e.g., [3]), although faster shifts can be induced under specific stimulus conditions [1]. We thus believe that the PSD obtained with the diffusive currents simulated here should be a realistic prediction of what one could observe experimentally under physiological conditions with large ECS concentration gradients.

In the current work, diffusive potentials arose due to large scale concentration gradients in the ECS bulk solutions. We note that these diffusive effects relate to a different phenomenon than the diffusion-evoked 1/*f*-filtering effects proposed by Bedard and Destexhe (with co-workers). These authors developed a mean-field description of neuronal tissue (comprising both membranes and ECS), and incorporated diffusion-evoked frequency-filtering effects in terms of a complex *Warburg impedance* [41–43, 89–91]. They argued that the Warburg effects arises due to highly localized diffusion processes in the membrane-near Debye-layers when charge is transferred from the intracellular to extracellular space [90, 91]. Traditionally, the Warburg impedance has been derived for complex interactions at interfaces between electrodes and electrolytes, where the chemical reactions necessary for transferring charge between the electrode surface and electrolyte requires a continuous, diffusion-dependent, reshuffling of local ions [92, 93]. The physical argument to why similar effects should take place close to neuronal membranes is presently unclear, and a complex conductivity, accounting for such possible effects of membrane-near filtering, was not included in the present model. In any case, such putative Warburg-type effects arising close to the membrane and the diffusion potentials evoked by large scale ECS concentration gradients describe different and complementary effects that ionic diffusion could have on the LFP, and are not *a priori* in contradiction.

#### Power spectrum of membrane source-generated potential (P2)

The realism of the membrane-current generated ECS potentials (P2), on the other hand, depend on whether neural population model was sufficiently detailed to generate ECS potentials that one would expect under realistic experimental conditions.

It is generally not trivial to construct models that reproduce experimentally recorded PSDs [17]. Previous studies have shown these to be sensitive to the distribution and balance between excitatory and inhibitory synapses on the neuronal membranes [33, 36]. Furthermore, the LFP is also likely to receive contributions from a large fraction of neurons that are not firing APs, but receiving synaptic input, so that they still participate in generating the low powers of the PSD [33]. In addition, different aspects of the LFP has been found to depend on neuronal morphology, subcellular distributions of membrane mechanisms, and the level of synchrony between neighboring neurons [18, 19, 33, 35, 94–96].

In the case of large deviances from baseline ECS concentrations, also glial buffering mechanisms [3, 16] and neuronal uptake mechanisms such as Na^+^/K^+^-exchangers [83] could constitute additional slow membrane currents that could influence the low frequencies of the LFP. Generally, such mechanisms also act to reduce concentration gradients in the ECS. Whereas the concentration gradients seen in the current simulations (not including uptake mechanisms) were realistic, such gradients would probably under most conditions require a higher neural activity level (P2) than in the current model, since the neuronal output would need to out-compete uptake mechanisms in order to generate ECS gradients.

Inhibitory synapses, inactive neurons, ion pumps and glial buffering mechanisms were not included in the model considered here. In a realistic scenario, it is likely that these mechanisms could enhance the membrane-current induced (P2) contribution to the lowest frequencies in the PSD compared to what we predicted in the current model (see below for further discussion on this).

#### Power spectrum of the full electrodiffusive model (P1)

The PSDs seen in ECS recordings is a highly complex topic. Generally, the frequency scaling is multifactorial, dependent on state (sleep/wake) [97], neural correlation/decorrelation [35, 95], size of active populations [35], morphology of nearby, active neurons [34, 98], specific activity (spiking frequency/synaptic input) of nearby neurons [33], and possibly by frequency filtering within the extracellular medium [41, 97] and diffusion along extracellular concentration gradients, as seen here.

If different causes for frequency scaling are linearly dependent (mathematically, this means that two processes can be expressed as a convolution), it leads to addition of the individual powers (exponents). For example, if an incoming spike train triggers synaptic currents, and both the underlying processes have a 1/*f*^2^ frequency scaling, the net scaling will be 1/*f*^4^. As indicated in Fig 9, the large-scale diffusion process considered in the current work is quite independent of other processes, and the power (exponent) from diffusion does not add to the powers (exponents) of the transmembrane sources. In this regard, the large-scale diffusion effects differ from the Warburg-type filtering effects hypothesized in some other works to take place in thin sheaths surrounding neuronal membranes [90, 91]. This implies that large-scale diffusion will will exhibit its characteristic 1/*f*^2^ frequency scaling, and will be visible in the PSD only in the frequency range where diffusion is the dominant process (when such a range exists). It also means that the presence of diffusion is not in conflict with observing an undisturbed power law generated by other processes (this would only imply that the other process dominates).

As we have argued above, the PSD predicted for the diffusive process (P3) should be physiologically realistic (for large concentration gradients), while the low-frequency components of the membrane-current induced PSD in our model (P2) may be an underestimation of what would be expected in a real system. If additional (slow) membrane mechanisms were included, we would expect the range of frequencies where diffusion dominated the PSD (and the crossing point between the red line (P2) and black line (P3) in Fig 9B3) to be shifted towards lower frequencies (i.e. lower that the 1–10 Hz found here, and in many cases, possibly to frequencies below the cut-off frequency used in LFP recordings). However, given the steepness (1/*f*^2^) of the diffusion-generated PSD, a cross-over frequency below which diffusion dominates the PSD, is still likely to occur, especially under conditions where we can expect large extracellular concentration gradients. By prediction, we would then expect to observe a diffusion evoked 1/*f*^2^ scaling for some low-frequency range of the PSD.

A 1/*f*^2^ scaling for low frequencies has indeed been observed in cerebral areas of human patients with epilepsy [99, 100], a pathological condition which is strongly associated with dramatic changes in ECS concentrations [101]. A similar scaling was found in slices from rat hippocampus when epileptic-seizure-like events were induced [102]. It should be noted, however, that also processes other than diffusion can give rise to a 1/*f*^2^ scaling of the PSD [41, 99], and that the 1/*f*^2^ scaling in one of the cited studies was originally explained by slow-wave state transitions between up/down states [99].

### Implication for current source density estimates

A common starting point for the estimation of the current-source density *CSD*(*x*, *y*, *x*) from the ECS potential *V*(*x*, *y*, *z*) is [37, 39]:

∇(σ∇V)=-CSD (1)

The left hand side is the divergence of the ECS currents, and an implicit assumption in this equation is that only electric currents driven by the electrical field is present in the ECS, i.e., solely Ohmic current densities given by *i*^*f*^ = −*σ*∇*V*. If also diffusive ECS currents were accounted for, the corresponding equation would be:

$$\nabla \left(\sigma \nabla V\right)-\nabla i^{d}=-CSD,$$ (2)

where the diffusive current density is a function of ionic concentrations in the ECS (see Methods, Eq 9). The use of Eq 1 for predicting the CSD could thus lead to a misinterpretation of diffusive ECS currents (if present in the real system) as neuronal current sources.

An example where experimental recordings seems to disagree with the standard CSD theory (Eq 1) was reported recently by Riera et al. [30] who found that the estimated instantaneous current-source density (CSD) from recorded ECS potentials did not sum to zero over the volume of the barrel column. According to the standard CSD-theory, this would indicate the presence of a non-zero current-source monopole on a mesoscopic (cell population) scale. The possible origin of these apparent current monopoles was later debated [30, 103–106]. A non-negligible diffusive source term, cf. Eq 2 could be one (of several) possible explanations of this discrepancy between experiments and original CSD-theory.

### Outlook

The model presented here was a simplified one, both in terms of using a 1D geometry and in terms of neglecting several neuronal and glial mechanisms that would likely contribute to the generation of the LFP. A future ambition is to expand this framework to a 3D model that also accounts for more of the complexity of neuronal tissue, and includes effects of neuronal and glial ionic uptake mechanisms (ion pumps). A 3D version of the KNP framework could ideally be combined with existing, comprehensive simulators of large neuronal networks such as the Blue Brain simulator [69]. We believe that such a framework would be very important for the field of neuroscience as it not only would be useful for exploring how diffusive currents can have an impact on ECS potentials, but also to simulate various pathological conditions related to ion-concentration dynamics in neural tissue [9, 13–15, 101, 107].

## Materials and Methods

From a method-development point of view, the main contribution of this work was the development of a hybrid scheme (Fig 1) for combining the NEURON-simulator [60] (used to simulate the dynamics of a neuronal population), with the KNP formalism [28, 57] (used to compute the dynamics of ion concentrations and the electrical potential in the ECS surrounding the neuron population). The two components used in this scheme are presented in further detail below.

### Kirchhoff-Nernst-Planck formalism for extracellular dynamics

What we have here coined the *Kirchhoff-Nernst-Planck formalism*, was originally developed for computing the intra- and extracellular dynamics of ion concentrations and the electrical potential during astrocytic K^+^ buffering [28]. In the current application, it was only applied in the ECS (the intracellular space was handled with the NEURON-simulator). For simplicity, we assumed that spatial variation only occurred in one spatial direction (*z*-direction), and thus that we had lateral homogeneity of all state variables.

#### Continuity equation

The KNP formalism represents a way of solving the continuity equation for the ionic concentrations ($c_{n}^{k}$ (mol/m^3^)), and is here derived for the system sketched in Fig 1B. The ECS is subdivided into a number of *N* = 15 subvolumes of length *l*_*c*_ and cross section area *A*_*c*_. Using data from cortex (as in [35]), we assume that the average surface area per neuron is about 300 *μ*m^2^, so that the ten neurons used in our simulations occupy a surface area *A*_*c*_ = 3000 *μ*m^2^. The vertical length of a subcompartments is set to *l*_*c*_ = 100 *μ*m, so that the neuron (having a vertical extension of slightly below 1300 *μ*m) occupy the interior 13 subvolumes.

In each subvolume *n*, the concentrations of all present ion species *k* are assumed to be known. Ions may enter the subvolume either via (i) transmembrane fluxes from neurons that exchange ions with the subvolume (*J*^*kM*^), (ii) diffusive fluxes between neighboring subvolumes (*J*^*kd*^), or (iii) field fluxes between neighboring subvolumes (*J*^*kf*^). The formalism computes the ECS fluxes (in mol/s), and can be applied together with any selection of neuronal sources. For now, we assume that the transmembrane fluxes *J*^*kM*^ for all ion species as well as the transmembrane capacitive current (which will be relevant below) are known (e.g., determined from a separate simulation using, e.g., the NEURON simulator [60], as we shall return to later). The continuity equation is (in discretized form):

$$\alpha A_{c}l_{c}\frac{\partial c_{n}^{k}}{\partial t}=J_{n}^{kM}+J_{n-1,n}^{kd}-J_{n,n+1}^{kd}+J_{n-1,n}^{kf}-J_{n,n+1}^{kf}$$ (3)

where we have used the notation that *J*_*n*−1,*n*_ denotes the flux from subvolume *n* − 1 to subvolume *n*. The extracellular fluxes are described by the Nernst-Planck equations [108]:

$$J_{n-1,n}^{kd}=-\frac{\alpha A_{c}}{l_{c}}\frac{D^{k}}{\lambda^{2}}\left(c_{n}^{k}-c_{n-1}^{k}\right),$$ (4)

and

$$J_{n-1,n}^{kf}=-\frac{\alpha A_{c}}{\psi l_{c}}\frac{z^{k}D^{k}}{\lambda^{2}}\frac{c_{n-1}^{k}+c_{n}^{k}}{2}\left(V_{n}-V_{n-1}\right),$$ (5)

where the factor *ψ* = *RT*/*F* is defined in terms of the gas constant (*R* = 8.314 *J*/(mol K)), the absolute temperature (*T*), and Faraday’s constant (*F* = 96,485C/mol).

We have used the porous medium approximation, characterized by the parameters *α* and λ [74]. The parameter *α* represents the fraction of the tissue volume being ECS, and we used the value *α* = 0.2 [4, 74]. The prefactor *αA*_*c*_*l*_*c*_ in eq 3 then equals the ECS volume of a subvolume *n*. The extracellular tortuosity λ represents miscellaneous hindrances to motion through neuronal tissue [4, 74, 109], and gives rise to a reduced effective diffusion constant ${\tilde{D}}^{k}=D^{k}/\lambda^{2}$ where *D*^*k*^ is the diffusion constant for ion species *k* in dilute solvents. We used the value λ = 1.6 [4], and standard values for the diffusion constants [110]: ${\tilde{D}}^{K}=1.96\times 10^{-9}m^{2}/s$, ${\tilde{D}}^{Na}=1.33\times 10^{-9}m^{2}/s$, ${\tilde{D}}^{Ca}$ = 0.71 × 10^−9^m^2^/s and ${\tilde{D}}^{X}=2.03\times 10^{-9}m^{2}/s$ (Here, X is an unspecified ion species (see below), for which we used the diffusion constant for Cl^-^).

We assume that the edge subvolumes (*n* = 1 and *n* = *N*) represent a background where ion concentrations remain constant. The continuity equation then governs the ion-concentration dynamics in all the *N* − 2 interior subvolumes. If we include a number *K* of different ion species, the continuity equation (Eq 3) for *n* = 2, 3, …, *N* − 1 and *k* = 1, 2, …, *K* gives us *K*(*N* − 2) conditions for the *K*(*N* − 2) ion concentrations $c_{n}^{k}$ in the *N* − 2 subvolumes where ion concentrations are dynamically changing. However, the continuity equation also includes *N* state variables for the potential *V*_*n*_ in all subvolumes (including the edges). We thus need *N* additional constraints to fully specify the system.

#### Derivation of extracellular potential

In the following, we derive expressions for the ECS potential (*V*_*n*_) based on the principle of Kirchhoff’s current law, and the assumption that the bulk solution is electroneutral [28]. To do this, we multiply the continuity equation (Eq 3) by *Fz*^*k*^, take the sum over all ion species *k*, and obtain the continuity equation for electrical charge:

$$\frac{\partial q_{n}}{\partial t}=I_{n}^{M}+I_{n-1,1}^{d}-I_{n,n+1}^{d}+I_{n-1,n}^{f}-I_{n,n+1}^{f}$$ (6)

Here, we have transformed fluxes/concentrations into electrical currents/charge densities by use of the general relations [108]:

$$I_{n}^{M}=F\sum_{k}\left(z^{k}J_{n}^{kM}\right),$$ (7)

$$q_{n}/\left(\alpha A_{c}l_{c}\right)=\rho_{n}=F\Sigma_{k}\left(z^{k}c_{n}^{k}\right),$$ (8)

$$I_{n-1,n}^{d}=F\Sigma_{k}\left(z^{k}J_{n-1,n}^{kd}\right)=-\frac{F\alpha A_{c}}{l_{c}}\Sigma_{k}\left(\frac{z^{k}D^{k}}{\lambda^{2}}\left(c_{n}^{k}-c_{n-1}^{k}\right))\right)$$ (9)

and

$$I_{n-1,n}^{f}=F\sum_{k}\left(z^{k}J_{n-1,n}^{kf}\right)=-\frac{\alpha A_{c}}{l_{c}}\sigma_{n-1,n}\left(V_{n}-V_{n-1}\right),$$ (10)

where *z*^*k*^ is the valence of ion species *k* and *F* is Faraday’s constant. In Eq 10, we also defined the conductivity (units (*Ωm*)^−1^)for currents between two subvolumes *n* − 1 and *n* as:

$$\sigma \left(n-1,n\right)=F\sum_{k}\left(\frac{D^{k}{\left(z^{k}\right)}^{2}}{\lambda^{2}\psi }\frac{c_{n-1}^{k}+c_{n}^{k}}{2}\right)$$ (11)

At time scales larger than nanoseconds, bulk solutions can be assumed to be electroneutral [111]. In our scheme, bulk electroneutrality implies that any net ionic charge entering an ECS subvolume must be identical to the charge that enters a capacitive neural membrane within this subvolume. This is also an implicit assumption in the cable equation (see, e.g., [28, 64, 108, 112]) upon which the NEURON simulator is based. With this assumption at hand, the continuity equation for charge (Eq 6) becomes useful for us, as it is governed by a constraint that we did not have at the level of ion concentrations (Eq 3). Electroneutrality in the bulk solution implies that the net charge entering an ECS subvolume (the time derivative of *q*_*n*_ in Eq 6) must be identical to the charge which accumulates at the neuronal membrane and gives rise to the neurodynamics. This means that the time derivative of *q*_*n*_ must be equal to the capacitive current that we know from the NEURON simulator:

$$\frac{\partial q_{n}}{\partial t}=-I_{n}^{cap}$$ (12)

Thus, *q*_*n*_ (in Eq 6) is not an independent state variable, but an entity given from the NEURON simulation (i.e., an input condition to the ECS). With this at hand, we can rewrite Eq 6) on the form:

$$-I_{n}^{cap}-I_{n}^{M}=I_{n-1,1}^{d}-I_{n,n+1}^{d}+I_{n-1,n}^{f}-I_{n,n+1}^{f}$$ (13)

We now see that Eq 13 is simply Kirchhoff’s current law, and states that the net current into an ECS volume *n* is zero, cf. Fig 1C. If we insert Eq 10 for *I*^*f*^, Eq 13 becomes:

$$\sigma_{n-1,n}V_{n-1}-\left(\sigma_{n-1,n}+\sigma_{n,n+1}\right)V_{n}+\sigma_{n,n+1}V_{n+1}=\frac{l_{c}}{\alpha A_{c}}\left(-I_{n}^{cap}-I_{n}^{M}-I_{n-1,1}^{d}+I_{n,n+1}^{d}\right)$$ (14)

We note that $I_{n}^{M}$ was defined as the net *ionic* transmembrane current (Eq 7), and that it does not include the capacitive current. We further note that Eq 14 for a subvolume (*n*) depends on the voltage levels in the two neighbouring subvolumes (*n* − 1 and *n* + 1), and thus only gives us *N* − 2 conditions, i.e., one for the *N* − 2 inferior volumes. We need two additional criteria for the edge subvolumes (*n* = 1 and *n* = *N*). As we may chose an arbitrary reference point for the voltage, we may take the first criterion to be:

$$V_{1}=0$$ (15)

As the second criterion, we impose a boundary condition stating that no net electrical current is allowed to pass between the subvolumes *n* = *N* − 1 and *n* = *N* (i.e. no net electrical current enters/leaves the system from/to the constant background). Since there may be a diffusive current between these two subvolumes ($c_{N-1}^{k}$ is not constant), this criterion implies that we must define *V*_*N*_ so that the field current is opposite from the diffusive current ($I_{N-1,N}^{d}+I_{N-1,N}^{f}=0$). If we insert for *I*^*f*^ (cf., Eq 10), this condition becomes:

$$\sigma_{N-1,N}\left(V_{N-1}-V_{N}\right)=\frac{l_{c}}{\alpha A_{c}}I_{N-1,1}^{d}$$ (16)

The conductivity (*σ*) and the diffusive currents (*I*^*d*^) are defined by ionic concentrations in the ECS, whereas we assumed that the neuronal output (*I*^*cap*^ and *I*^*M*^) was known. Eqs 14–16 thus give us *N* equations for the *N* voltage variables *V*_*n*_. In matrix form, we can write the system of equations (Eqs 14–16) as:

AV=b, (17)

where **V** is a vector containing the potential *V*_*n*_ in all *N* subvolumes, and **b** is a vector with *N* elements given by:

$$b_{n}=\left\{\begin{array}{cc} 0 & \text{for}\;n=1\\ \frac{l_{c}}{\alpha A_{c}}\left(-I_{n}^{cap}-I_{n}^{M}-I_{n-1,1}^{d}+I_{n,n+1}^{d}\right) & \text{for}\;n=1,2,...,N-1\\ \frac{l_{c}}{\alpha A_{c}}\left(I_{N-1,1}^{d}\right) & \text{for}\;n=N \end{array}\right.$$ (18)

The *N* × *N* matrix *A*:

$$A=\left(\begin{array}{cccccc} a_{1,1} & a_{1,2} & 0 & 0 & \cdots & 0\\ a_{2,1} & a_{2,2} & a_{2,3} & 0 & \cdots & 0\\ 0 & a_{3,2} & a_{3,3} & a_{3,4} & \cdots & 0\\ \vdots & \ddots & \ddots & \ddots & \ddots & \vdots \\ 0 & 0 & \cdots & a_{N-1,N-2} & a_{N-1,N-1} & a_{N-1,N}\\ 0 & 0 & \cdots & 0 & a_{N,N-1} & a_{N,N} \end{array}\right)$$ (19)

is a tridiagonal matrix. The diagonal above the main diagonal is given by:

$$a_{n,n+1}=\left\{\begin{array}{cc} 0 & \text{for}\;n=1\\ \sigma_{n,n+1} & \text{for}\;n=2,3,...,N-1 \end{array}\right.$$ (20)

The diagonal below the main diagonal is given by:

$$a_{n,n-1}=\left\{\begin{array}{cc} \sigma_{n,n+1} & \text{for}\;n=2,3,...,N \end{array}\right.$$ (21)

The main diagonal is given by:

$$a_{n,n}=\left\{\begin{array}{cc} 1 & \text{for}\;n=1\\ -\left(\sigma (n-1,n)+\sigma (n,n+1)\right) & \text{for}\;n=2,3,...,N-1\\ -\sigma (N-1,N) & \text{for}\;n=N \end{array}\right.$$ (22)

For each time step in the simulation, we can determine *V*_*n*_ by solving the algebraic equation set:

$$V=A^{-1}b,$$ (23)

where *A*^−1^ is the inverse of the matrix *A*.

When we ran simulations where diffusion was not included, *J*^*d*^ was simply set to zero in the continuity equation (Eq 3), and *I*^*d*^ was set to zero in the equation where the ECS potential is derived (Eq 18).

#### Initial conditions

As initial conditions, we assumed that all ECS volumes were at potential *V*_*n*_ = 0. The initial ion concentrations were also identical in all ECS subvolumes. We used *c*^*K*0^ = 3 mM, *c*^*Na*0^ = 150 mM, *c*^*Ca*0^ = 1.4 mM. These ion concentrations are quite typical for cerebrospinal fluid [113]. To obtain an initial charge density of zero in the bulk solution, we computed that the initial concentration for the unspecified anion should be *c*^*X*0^ = 155.8 mM: With this value, we get that local charge density *ρ*/*F* = ∑*z*^*k*^ *c*^*k*0^ = (1 × 3 + 1 × 150 + 2 × 1.4 − 1 × 155.8) mM = 0. This value for *c*^*X*0^ is close to typical ECS concentrations for Cl^-^ [113], and the unspecified ion X^-^ can be seen as essentially taking the role that Cl^-^ has in real systems.

#### Power spectrum analysis

The power spectra (Fig 8) were computed with the fast Fourier-transform in MATLAB (http://se.mathworks.com/) and filtered to give one value per 0.1 log unit of the frequency.

### Neuronal population dynamics

In the current work, the KNP formalism was used to predict the extracellular ion-concentration dynamics and electrical potential surrounding a small population of ten pyramidal cells. The neural simulation used in this study was briefly introduced in the Results section, but is presented in further detail here.

#### Pyramidal cell model

As neural model, we used the thick-tufted layer 5 pyramidal cell model by Hay et al. [62], which was implemented in the NEURON simulation environment [60]. The model was morphologically detailed (it had 196 sections, each of which we divided into 20 segments), and had a vertical extension of slightly less than 1300 *μ*m from the tip of the basal dendrite to the tip of the apical dendrites. It contained ten active ion channels with different distributions over the somatodendritic membrane, including two Ca^2+^-channels (*i*^*CaT*^, *i*^*CaL*^), five K^+^-channels (*i*^*KT*^, *i*^*KP*^, *i*^*SK*^, *i*^*Kv*3.1^, *i*^*M*^) and two Na^2+^-channels (*i*^*NaT*^, *i*^*NaS*^). In addition, it included a non-specific ion channel (*i*^*h*^) and the non specific leakage current *i*^*leak*^. The neuron had a membrane capacitance of 1 *μ*F/cm^2^ in the soma, and 2 *μ*F/cm^2^ in the dendrites, and leak conductances ranging between 0.0325 and 0.0589 mS/cm^2^ over the somatodendritic membrane. We refer to the original publication for further model details [62].

#### Synapse model

The neurons received Poissonian input trains through 10,000 synapses per neuron, a typical number for cortical neurons [114]. Each synapse had a mean input spike rate of 5 Hz. The synapses were uniformly distributed across the membrane so that the expected number of synapses in a segment was proportional to its membrane area. A population of ten neurons was simulated by running 10 independent simulations with the same neural model. The synapse distribution and spike trains were regenerated for each of the independent simulations (but with the same statistics in each case).

The synapses were modelled as *α*-shaped synaptic conductances:

$$I\left(t\right)=\left\{\begin{array}{c} g_{max}\left(t-t_{0}\right)/\tau \;\exp\left[1-\left(t-t_{0}\right)/\tau \right],\;\text{when}\;t\geq t_{0}\\ 0,\;\text{when}\;t<t_{0} \end{array}\right.,$$ (24)

where *t*_0_ represents the time of onset. The time constant was set to *τ* = 2.0 ms. The maximal conductances of the synapses were set to *g*_*max*_ = 0.042 nS. For this value, the input evoked an average single-neuron action-potential (AP) firing rate of about 5 APs per second, which is a typical firing rate for cortical neurons [35, 63].

#### Population output to ECS

The spatial extension of the cell morphology [62] was such that the maximal spatial distance between two segments (from tip of basal dendrite to tip of apical dendrite) was less than 1300 *μ*m. We therefore considered a tissue depth of 1500 *μ*m, and subdivided it into *N* = 15 ECS subvolumes (depth intervals) of length *l*_*c*_ = 100 *μ*m, so that the neurons occupied the interior 13 subvolumes. Each neural segment was assigned as belonging to a particular subvolume *n*, determined by the spatial location of the segment midpoint. In the setup, the soma was placed in subvolume *n* = 3, the basal dendrites were in subvolumes *n* = 2, 3 and 4, and the apical dendrites were in subvolumes *n* = 3, …, 14. The multicompartmental model also included a short axon, which was, however, not based on the reconstruction and hence had no fixed coordinates. We assigned the axonal segments into the same subvolume as the soma, *n* = 3. The boundary subvolumes 1 and 15 contained no neural segments (see Fig 1A).

The transmembrane current density ($i_{seg}^{kM}$) of ion species *k* is available in the NEURON simulation environment. It was multiplied by the surface area of the segment (*A*_*seg*_) to get the net current, and divided by Faraday’s constant (*F*) to get a net ion flux with units mol/s. During the neural simulation, we grouped all currents that were carried by a specific ion species into the net transmembrane influx/efflux of this ion species. We assumed that all non-specific currents, including the synaptic currents (*i*^*leak*^, *i*^*h*^, *i*^*syn*^) were carried by a non-specific anion that we denoted X^-^. In this way we could compute the net efflux of each ion species into a subvolume *n*:

$$\begin{array}{c} J_{n}^{CaM}=\frac{1}{2F}\sum_{seg}\left(i_{seg}^{CaT}+i_{seg}^{CaL}\right)A_{seg}\\ J_{n}^{NaM}=\frac{1}{F}\sum_{seg}\left(i_{seg}^{NaT}+i_{seg}^{NaS}\right)A_{seg}\\ J_{n}^{KM}=\frac{1}{F}\sum_{seg}\left(i_{seg}^{KT}+i_{seg}^{KP}+i_{seg}^{SK}+i_{seg}^{Kv3.1}+i_{seg}^{M}\right)A_{seg}\\ J_{n}^{XM}=-\frac{1}{F}\sum_{seg}\left(i_{seg}^{leak}+i_{seg}^{h}+i_{seg}^{syn}\right)A_{seg} \end{array}$$ (25)

Here, the sum was taken over all neural segments (*seg*) of all 10 neurons contained in subvolume *n*. The factor 2 in the denominator in the expression for $J_{n}^{CaM}$ was due to *Ca*^2+^ having valence 2, and the negative sign in the expression for $J_{n}^{XM}$ was due to *X*^−^ having valence -1. We also kept track of the (non-ionic) capacitive currents, as required by the electrodiffusive formalism (Eq 14):

$$I_{n}^{cap}=\sum_{seg}i_{seg}^{cap}A_{seg}$$ (26)

The intracellular dynamics was directly adopted from the original model [62]. Transmembrane currents there had no effect on intracellular ion concentrations, except for Ca^2+^ concentration, which was modelled to account for Ca^2+^ dependent K^+^ channels.

For technical reasons (concerning memory usage), 84 s of output from a single neuron was generated in the following way: First, we ran ten simulations, each producing 10 s of activity. Next, we removed the initial 1.6 s of all the ten simulations, to remove the transient neuronal activity observed initially in the simulations, leaving us with ten 8.4 s time series. Finally, these were used as successive output periods from a single neuron, and combined into a total 84 s time series.

### Implementation

Simulations on the pyramidal cell model by [62] was run the NEURON/Python simulation environment [60]. The ECS dynamics was computed separately with the KNP formalism, using the neuronal output/input as an external input time series. The KNP model was implemented in MATLAB (http://se.mathworks.com/). The MATLAB code (along with the neuronal input time series) will be made publicly available at ModelDB (http://senselab.med.yale.edu/modeldb).

## Supporting Information

S1 Appendix The diffusion-evoked 1/*f*^2^ power law in *V*. (PDF)

S2 Appendix The Goldman-Hodgkin-Katz (GHK) equation. (PDF)
