# Correction: Effect of Ionic Diffusion on Extracellular Potentials in Neural Tissue

**DOI:** 10.1371/journal.pcbi.1006050

**Published:** 2018-03-07

**Authors:** The PLOS Computational Biology Staff

The funding statement for this article should read as follows:

"Funding: This project was funded by the European Union Seventh Framework Programme (FP7/2007-2013) under grant agreement 604102 (Human Brain Project, HBP), and the Research Council of Norway (NFR, through ISP & Digital Life project “Digibrain” 248828). The funders had no role in study design, data collection and analysis, decision to publish, or preparation of the manuscript."
