# Supplementary material for: Effect of Ionic Diffusion on Extracellular Potentials in Neural Tissue
Source: PLoS Comput Biol. 2016 Nov 7;12(11):e1005193. doi: 10.1371/journal.pcbi.1005193 (PMC5098741; doi:10.1371/journal.pcbi.1005193)
Supplement: S1 Appendix — (PDF) [file pcbi.1005193.s001.pdf]

S1 Appendix A

We here show analytically that in the absence of neuronal sources ECS diffusion should give rise to an dynamics in the ECS potential described by a $1/f^2$ power law (i.e., as predicted in Fig. 9 in main text).

The KNP formalism requires an equilibrium between diffusive and field currents, which means that we have (at all times):

$$\frac{\partial V}{\partial x} = -\frac{F}{\sigma \lambda^2} \sum_k z^k D^k \frac{\partial c^k}{\partial x}, \tag{1}$$

where we for simplicity have assumed that the electrical conductivity σ is constant. If we integrate this with respect to x we see that:

$$V \sim \sum_k z^k D^k c^k \tag{2}$$

We thus expect V to follow the same power law as c^k.

In the following, we only consider one ion species with concentration $c(x,t)$ and diffusion constant D. We make the simplifying assumption that c in the absence of neuronal sources only changes due to diffusion (i.e., ion-concentration changes due to electrical migration are neglected), so that the temporal development of c is determined by the diffusion equation:

$$\frac{\partial c}{\partial t} = \frac{D}{\lambda^2} \frac{\partial^2 c}{\partial x^2} \tag{3}$$

We note that these simplifications might not *a priori* be justified. However, we show below that they do lead to the $1/f^2$ power law in the diffusion potential that we saw in the simulations (see Fig. 9 in main text). For simplicity, we let $c(x,t)$ denote the deviance from baseline concentration, and solve Eq. 3 on an interval $0 < x < L$ and $0 < t < T$ with fixed boundary conditions $c(0,t) = c(L,t) = 0$. The solutions of this system can be found using separation of variables, so that we may write $c(x,t) = f(x)g(t)$ (see e.g., chapter 13 in [1]):

$$\frac{\lambda^2}{D} \frac{dg/dt}{g} = \frac{d^2 f/dx^2}{f} = -p^2, \tag{4}$$

where $-p^2$ is a negative constant (it can be shown that only negative constants give acceptable solutions).

The general solution for $f(x)$ is:

$$f(x) = a \sin(px) + b \cos(px) \tag{5}$$

The boundary condition $f(0) = 0$ requires that $b = 0$, while the boundary condition $f(L) = 0$ requires that $pL = \pi m$, for some integer m, or:

$$p_m = \pi m / L, \tag{6}$$

so that the valid solutions are:

$$f_m(x) = a_m \sin(p_m x) \tag{7}$$

Since they are related by Eq. 4, each of the solutions for $f_m(x)$ has a corresponding solution for $g_m(t)$:

$$g_m(t) = k_m e^{-p_m^2 Dt/\lambda^2}, \tag{8}$$

where k_m is a constant of integration.

Linear combination of solutions $c_m = f_m g_m$ are also solutions, so that we may generally write:

$$c(x,t) = \sum_{m=1}^{\infty} A_m \sin(p_m x) \exp(-p_m^2 Dt/\lambda^2) \tag{9}$$

where $A_m = a_m k_m$. All solutions on the form in Eq. 9 satisfy the diffusion equation, but only one realization of the coefficients A_m will satisfy a given initial concentration $c(x,0)$. It can be shown that the coefficients A_m are given by:

$$A_m = 2\int_0^L c(x,0)\sin(p_m x)dx \tag{10}$$

Our objective was to show that the temporal development of c had a power spectrum $\sim 1/f^2$, or an amplitude spectrum $\sim 1/f$. To show this, we Fourier expand the temporal part ($g_m(t) = \exp(-p_m^2 Dt/\lambda^2)$) of Eq. 9 at some time interval $[0,T]$:

$$g_m(t) = \alpha_{m0} + \sum_{n=1}^{N} (\alpha_{mn}\cos(\omega_n t) + \beta_{mn}\sin(\omega_n t)) \tag{11}$$

with $\omega_n = 2\pi n/T$. The coefficients are given by:

$$\alpha_{m0} = \frac{1}{T}\int_0^T g_m(t)dt \tag{12}$$

$$\alpha_{mn} = \frac{2}{T}\int_0^T g_m(t)\cos(\omega_n t)dt \tag{13}$$

$$\beta_{mn} = \frac{2}{T}\int_0^T g_m(t)\sin(\omega_n t)dt \tag{14}$$

and become:

$$\alpha_{m0} = \frac{1 - e^{-p_m^2 DT/\lambda^2}}{p_m^2 DT/\lambda^2} \tag{15}$$

$$\alpha_{mn} = \frac{2(1 - e^{-p_m^2 DT/\lambda^2})}{T}\frac{p_m^2 D}{(p_m^2 D/\lambda^2)^2 + (\omega_n)^2} \tag{16}$$

$$\beta_{mn} = \frac{2(1 - e^{-p_m^2 DT/\lambda^2})}{T}\frac{\omega_n}{(p_m^2 D/\lambda^2)^2 + (\omega_n)^2}. \tag{17}$$

Here α_{m0} is a constant (DC) term, which will not influence the power law in the PSD. We see that for frequencies that fulfill

$$p_m^2 D/\lambda^2 \ll \omega_n = 2\pi f_n \tag{18}$$

the coefficients α_{mn}, which will be proportional to $1/\omega^2$, will be small compared to β_n, which will be proportional to $1/\omega$. Thus, the β_n terms will dominate in Eq. 11. In this case, $g_m(t)$ and thus also the concentration c and amplitude of the extracellular potential V will follow a $1/f$ power law. Thus the power of this potential, corresponding to the squared amplitude of the potential, will follow a $1/f^2$ power law.

If we insert for p_m, the criterium in Eq. 18 implies that:

$$f_n \gg \pi m^2 D/(2L^2\lambda^2) \tag{19}$$

If we insert a typical value for the diffusion constant $D/\lambda^2 = 10^{-9}\mathrm{m}^2/\mathrm{s}$ and system length $L = 1.5 \times 10^-3$ m, we see that we can expect a $1/f^2$ power law for frequencies satisfying:

$$f \gg 0.6 \times 10^{-3} m^2 \,\mathrm{Hz} \tag{20}$$

According to Eq. 20, the spatial ion-concentration gradients (set by m) determine the frequency range that follows an $1/f^2$ power law. In our system, where concentrations varied on the length scale L (sf. Fig. 5 im main text), and we thus expect the long-wave components to dominate (i.e., we expect the coefficients A_m with small values of m to dominate the solution (Eq. 9). However, even for values as high as $m = 10$, the left hand side of Eq. 20 remains smaller (0.06 Hz) than the typical lower LFP cutoff frequencies in experiments ($f \sim 0.1$ Hz). Eq. 20 thus predicts that the in the relevant frequency range for the ECS diffusion, the PSD should follow an $1/f^2$ power law, as we also saw in our simulations (Fig. 9 in main text).

1 References

References

1. Boas ML. Mathematical methods in the physical sciences. Wiley; 2006.
