# Supplementary material for: Effect of Ionic Diffusion on Extracellular Potentials in Neural Tissue
Source: PLoS Comput Biol. 2016 Nov 7;12(11):e1005193. doi: 10.1371/journal.pcbi.1005193 (PMC5098741; doi:10.1371/journal.pcbi.1005193)
Supplement: S2 Appendix — (PDF) [file pcbi.1005193.s002.pdf]

S2 Appendix B

The Goldman-Hodgkin-Katz (GHK) equation for the equilibrium potential has the general form:

$$V = \frac{-RT}{F} \ln y \tag{1}$$

where y is the quadratic formula

$$y = \frac{-b + \sqrt{b^2 - 4ac}}{2a}. \tag{2}$$

Here, the parameters a, b and c are functions of ion concentrations in two compartments A and B. In the most standard form of the GHK-equation, only monovalent ion species are considered. When also Ca^{2+} is included, the equation has the form [1]:

$$a = 4P^{Ca}c^{CaA} + P^{K}c^{KA} + P^{Na}c^{NaA} + P^{ClA}c^{ClB} \tag{3}$$

$$b = P^{K}c^{KA} + P^{Na}c^{NaA} - P^{K}c^{KB} - P^{Na}c^{NaB} + P^{Cl}c^{ClB} - P^{Cl}c^{ClA} \tag{4}$$

$$c = -(4P^{Ca}c^{CaB} + P^{K}c^{KB} + P^{Na}c^{NaB} + P^{Cl}c^{ClA}) \tag{5}$$

Commonly, A and B are taken to represent the inside and outside of a permeable membrane, and the P^k represent the membrane permeability for ion species k. However, A and B may equally well represent two arbitrary compartments (e.g., two different depth layers in Fig. 1 in main text), and the permeability between the two compartments can be taken to be $P^k = D^k/(\lambda^2 l_{AB})$. The distance ($l_{AB}$) between A and B occurs both in the numerator and denominator of eq. 2, and will not influence V.

1 References

References

1. Sprangler S. Inverse current source density method in two dimensions: inferring neural activation from multielectrode recordings. The Alabama Journal of Medical Sciences. 2972;9:218–23.
